# Safety and immunogenicity of a conjugate vaccine candidate against *Salmonella enterica* serovars Typhi and Paratyphi A in healthy adults in Europe: a phase 1 randomised controlled trial

**DOI:** 10.1016/S1473-3099(25)00730-3

**Published:** 2026-06

**Authors:** Ilse De Coster, Mohammad AbdelGhany, Eleanna Sarakinou, Chiara Fineschi, Elisa Marchetti, Rita La Gaetana, Simona Nigro, Martina Carducci, Luisa Massai, Valentino Conti, Omar Rossi, Giulia Luna Cilio, Alimamy Serry-Bangura, Pietro Tessitore, Pierre Van Damme, Kanchanamala Withanage, Francesca Micoli, Francesco Berlanda Scorza, Simona Rondini, Usman N Nakakana, Ashwani Kumar Arora

**Affiliations:** a Centre for the Evaluation of Vaccination, Antwerp, Belgium; b GSK Vaccines Institute for Global Health, Siena, Italy; c GSK Vaccines, Siena, Italy; d Gates Foundation, Seattle, WA, USA

## Abstract

**Background:**

Enteric fever caused by *Salmonella enterica* serovars Typhi and Paratyphi A remains a major concern. No vaccines are licensed against *Salmonella* Paratyphi A. We aimed to assess the safety and immunogenicity of an investigational conjugate vaccine against *Salmonella* Typhi and Paratyphi A (Vi-CRM_197_+O:2-CRM_197_).

**Methods:**

In this observer-masked, randomised, controlled, dose-escalation, single-centre, phase 1 trial done during Nov 28, 2022, to April 2, 2024, at the Centre for Evaluation of Vaccination in Belgium, healthy adults (aged 18–50 years) were randomly assigned (2:1 or 2:2:1 across different steps using sealed envelopes following a randomisation schedule generated by an independent statistician) to receive two intramuscular doses (on day 1 and day 169) of one of four Vi-CRM_197_+O:2-CRM_197_ formulations (low dose or full dose, with or without aluminium hydroxide) or a control vaccine (Vi capsular polysaccharide vaccine and diphtheria toxoid-tetanus toxoid-acellular pertussis vaccine for first and second dose). The primary outcome was vaccine safety (solicited events during 7 days and unsolicited adverse events during 28 days after vaccination, serious adverse events [SAEs], and adverse events or SAEs leading to study withdrawal or withholding of further study intervention administration from day 1 to day 197, and deviations from normal or baseline laboratory test values 7 days after vaccination). Secondary outcomes included long-term vaccine safety (SAEs and adverse events or SAEs leading to study withdrawal from day 197 to day 337), and immunogenicity, including anti-Vi and anti-O:2 IgG antibody geometric mean concentrations and geometric mean ratios (GMRs) by ELISA, and seroresponses (percentages of participants with anti-Vi IgG concentrations ≥4·3 μg/mL and ≥2·0 μg/mL; ≥4-fold anti-O:2 IgG concentration increase from baseline) at day 1 (as applicable), day 29, day 169, day 176, and day 197. Safety analyses were done on the solicited safety set, unsolicited safety set, and the exposed set. The primary immunogenicity analysis was done on the per-protocol set defined by timepoint. The trial is registered with ClinicalTrials.gov (NCT05613205) and gsk-studyregister.com (205480), and is completed.

**Findings:**

96 participants were randomly assigned, 12 to each low-dose group, 24 to each full-dose group, and 24 to the control group. The incidence of solicited administration-site events (mostly pain) ranged from six (50% [95% CI 21·1–78·9]) of 12 participants in the low-dose without aluminium hydroxide group to 23 (96% [78·9–99·9]) of 24 in the full-dose with aluminium hydroxide group, versus 22 (92% [73·0–99·0]) of 24 in the control group. Solicited systemic events (mostly fatigue, headache, and myalgia) ranged from eight (67% [34·9–90·1]) of 12 in the low-dose groups to 20 (83% [62·6–95·3]) of 24 in the full-dose with aluminium hydroxide group, versus 21 (88% [67·6–97·3]) of 24 in the control group. The incidence of unsolicited adverse events (mostly nasopharyngitis) ranged from seven (58% [27·7–84·8]) of 12 in the low-dose without aluminium hydroxide group to ten (83% [51·6–97·9]) of 12 in the low-dose with aluminium hydroxide group, versus 14 (58% [36·6–77·9]) of 24 in the control group. Most safety laboratory results were within reference ranges. No SAEs occurred. After dose 1 (ie, at day 29), full-dose without aluminium hydroxide and full-dose with aluminium hydroxide induced the highest anti-Vi IgG responses (GMR 53·01 [95% CI 31·94–87·99] and 31·55 [18·74–53·11], respectively) versus control (4·50 [2·93–6·90]). Full-dose without aluminium hydroxide and low-dose without aluminium hydroxide induced the highest anti-O:2 IgG responses after dose 1 (GMR 162·61 [91·17–290·04] and 114·19 [44·83–290·86], respectively), versus control (1·27 [1·02–1·60]). 89–100% and 82–100% of participants (lowest percentages for low-dose with aluminium hydroxide) had anti-Vi IgG ≥4·3 μg/mL at day 29 (in initially seronegative participants) and ≥4-fold anti-O:2 IgG increase from baseline, respectively, versus 13 (54% [95% CI 32·8–74·4]) and one (4% [0·1–21·1]) of 24 participants in the control group, respectively. The second dose did not boost the responses.

**Interpretation:**

Vi-CRM_197_+O:2-CRM_197_ formulations did not raise safety concerns and showed immunogenicity with a single dose, supporting further clinical assessment of the full-dose without aluminium hydroxide in target populations (infants and older age groups) in endemic regions.

**Funding:**

Wellcome Trust.

## Introduction

*Salmonella enterica* serovars Typhi and Paratyphi A cause potentially fatal typhoid and paratyphoid fever, collectively known as enteric fever.^1^ In 2021, approximately 7·15 million enteric fever cases (93 300 deaths) were attributable to *Salmonella* Typhi and 2·17 million (14 300 deaths) to *Salmonella* Paratyphi A.^2^, ^3^

*Salmonella* Typhi has been included in the WHO bacterial high priority pathogens list since 2017.^4^ The emergence of typhoid resistant to all the recommended antibiotics for typhoid fever in Asia poses additional concerns, stressing the need for novel vaccines to combat such strains of *Salmonella* Typhi.^5^

Several vaccines against *Salmonella* Typhi are recommended by WHO, including typhoid conjugate vaccines, such as Vi polysaccharide-tetanus toxoid vaccines (ie, Typbar TCV, Bharat Biotech International, Hyderabad, India; and ZyVac TCV, Zydus Lifesciences, Ahmedabad, India), Vi polysaccharide-diphtheria toxoid (and its variants) vaccines (ie, Vi-CRM_197_; TYPHIBEV, Biological E, Hyderabad, India; and SKYTyphoid, SK Bioscience, Andong-si, South Korea), a live-attenuated oral Ty21a vaccine (ie, Vivotif, Bavarian Nordic, Hellerup, Denmark), and two non-conjugated Vi capsular polysaccharide vaccines (ie, Typhim Vi, Sanofi Pasteur, Lyon, France; and Typherix, GSK, Rixensart, Belgium).^6^ Typhoid conjugate vaccines can be administered to children aged 2 years or younger and induce stronger and more durable immunity compared with other typhoid vaccines, and are thus recommended by WHO.^7^ The efficacy of typhoid conjugate vaccines has been shown in several clinical trials; a single dose of Vi polysaccharide-tetanus toxoid vaccine showed a 78% efficacy over 4 years in children aged 9 months to 12 years in a phase 3 trial,^8^ and a pooled efficacy of 85% 1 year after vaccination in children aged 6 months to 16 years in a meta-analysis.^9^

**Research in context**

**Evidence before this study**

We carried out an extensive search of PubMed and ClinicalTrials.gov from inception using the search terms “typhoid conjugate vaccine”, “paratyphoid A vaccine”, and “Salmonella Paratyphi A”, up to the cutoff date of June 30, 2025, without any restrictions on publication dates or language. We retrieved seven relevant clinical studies related to: the outdated combined typhoid and paratyphoid A and B vaccine, which targets *Salmonella enterica* serovar Typhi and *S enterica* serovars Paratyphi A and B but is no longer in use; the *Salmonella* Paratyphi A conjugate vaccine, SPA-TT, which was tested in phase 1 and phase 2 studies showing an acceptable safety profile and substantial immune responses against the *Salmonella* Paratyphi A O-specific lipopolysaccharide 6 weeks after the first dose in children, adolescents, and adults, with no boosting effect of the second dose; the oral live-attenuated *Salmonella* Paratyphi A CVD 1902 vaccine, which elicited humoral and cellular immune responses against the *Salmonella* Paratyphi A-lipopolysaccharide in a phase 1 study in healthy adults and in a controlled human infection model, enabling the planned phase 1/2 trial (results not published); the combination vaccine Entervax, composed of Typhi ZH9 and an engineered derivative containing key antigens from *Salmonella* Paratyphi A (referred to as ZH9PA), which completed testing in a phase 1 study (results not published); and a bivalent conjugate vaccine against *Salmonella* Typhi and *Salmonella* Paratyphi A (Sii-PTCV), which in a phase 1 study elicited an immune response to both typhoid and paratyphoid antigens with no safety concerns identified. The paratyphoid component of the candidate vaccine against typhoid and paratyphoid fever was previously characterised in preclinical studies in mice and rabbits providing guidance for optimal vaccine design and induced a functional immune response in rabbits.

**Added value of this study**

We report findings from a phase 1 study to assess the safety and immunogenicity of another bivalent conjugate vaccine candidate against *Salmonella* Typhi and *Salmonella* Paratyphi A, Vi-CRM_197_+O:2-CRM_197_, in healthy adults in Europe. Compared with the previous phase 1 study with Sii-PTCV, we tested different vaccine formulations—ie, low-dose formulations (containing 5 μg of each Vi and O:2 antigen) and full-dose formulations (containing 25 μg of each antigen) with or without aluminium hydroxide adjuvant, and a different carrier protein (*Corynebacterium diphtheriae* CRM_197_) was used. All formulations were well tolerated by the participants, and no serious adverse events were reported. The first dose of all vaccine formulations induced robust immune responses at 1 month after vaccination to both Vi and O:2 antigens; in participants in the control group who received the currently licensed monovalent typhoid polysaccharide vaccine (Vi capsular polysaccharide vaccine), no response to the paratyphoid A component was observed. There was no boosting effect of the second dose. Full-dose without aluminium hydroxide induced a numerically higher or similar humoral response against *Salmonella* Paratyphi A than the adjuvanted formulation, with an anticipated favourable benefit–risk profile.

**Implications of all the available evidence**

Enteric fever due to *Salmonella* Typhi and *Salmonella* Paratyphi A remains a major health concern, especially in low-income and middle-income countries. Moreover, the burden of paratyphoid fever has been increasing, particularly in Asia, accounting for up to 25% of all enteric fever cases. The increasing roll-out of the monovalent typhoid conjugate vaccines might open a niche for *Salmonella* Paratyphi A serovar replacement. Therefore, bivalent vaccines against *Salmonella* Typhi and *Salmonella* Paratyphi A are urgently needed. The results of this study support further clinical assessment of the full-dose without aluminium hydroxide vaccine formulation for the prevention of both typhoid and paratyphoid enteric fever in infants and older age groups in endemic regions.

The burden of *Salmonella* Paratyphi A (relative to *Salmonella* Typhi) has been increasing, particularly in Asia;^10^ additionally, multidrug-resistant strains pose a substantial challenge to the treatment of paratyphoid fever.^11^ Yet, no vaccine against *Salmonella* Paratyphi A is currently available. Therefore, a bivalent conjugate vaccine for typhoid and paratyphoid fever would be valuable to prevent enteric fever in high-burden settings.

The virulence factor of *Salmonella* Typhi, a Vi capsular polysaccharide, considered the key antigen in typhoid conjugate vaccines, is absent in *Salmonella* Paratyphi A. The O-antigen portion of lipopolysaccharide (O:2), a key virulence factor of *Salmonella* Paratyphi A, has been proposed as target antigen for vaccines in development against paratyphoid fever.^10^

Here, we report the safety and immunogenicity results of a first-in-human study of a novel conjugate vaccine candidate against *Salmonella* Typhi and *Salmonella* Paratyphi A (Vi-CRM_197_+O:2-CRM_197_) combining the licensed Vi-CRM_197_ with the *Salmonella* Paratyphi A O:2 polysaccharide linked to the carrier protein cross-reacting material 197 (CRM_197_), and previously assessed in two pre-clinical studies.^12^, ^13^

## Methods

### Study design

This observer-masked, randomised, controlled, single-centre, dose-escalation, phase 1 study was done between Nov 28, 2022, and April 2, 2024, at the Centre for Evaluation of Vaccination (University of Antwerp, Antwerp, Belgium).

The study was done in accordance with the study protocol, the ethical principles derived from international guidelines including the Declaration of Helsinki and Council for International Organizations of Medical Sciences International Ethical Guidelines, the International Council for Harmonization Good Clinical Practice guideline, and applicable laws and regulations. The study was approved by the Independent Ethics Committee of the University of Antwerp (project ID 3815 - EudraCT 2021–002029–19).

The study is registered on ClinicalTrials.gov (NCT05613205) and gsk-studyregister.com (205480), and is complete. The study protocol (including the protocol amendments) and the statistical analysis plan are available online.

### Participants

Study participants were healthy adults aged 18–50 years at the time of the first study intervention administration who provided written informed consent. This population was selected since *Salmonella* Typhi and Paratyphi A are not endemic in this region, and as a low-risk population for the first-in-human assessment of vaccine safety. Race and biological sex data (male or female as options) were self-reported. The main exclusion criteria included significant medical conditions, immunodeficiency, use of immunosuppressants, previous typhoid vaccination, and recent travel to endemic areas. Eligibility criteria are detailed in the appendix (pp 3–4).

### Randomisation and masking

Participants were randomly assigned into seven study groups in three steps. In step 1a, participants were assigned in a 2:1 ratio to the low-dose without aluminium hydroxide group or the control group; in step 1b, participants were assigned in a 2:1 ratio to the low-dose with aluminium hydroxide group or the control group. In step 2, participants were assigned in a 2:2:1 ratio to the full-dose without aluminium hydroxide group, the full-dose with aluminium hydroxide group, or the control group (appendix pp 5–6).

Participants were sequentially assigned an identification number and randomly assigned using sealed envelopes according to a randomisation schedule generated by an independent statistician before study start.

The study was observer-masked until the first interim analysis done 28 days after dose 1. During this period, participants, the investigator, those assessing outcomes, and those analysing the data were masked to group assignments (appendix pp 5–7). Following the interim analysis and until the study end, no individual participant listings containing randomisation information were available. To ensure transparency and acknowledge the potential bias introduced by potential indirect unmasking for participants reporting unique safety events in group-unmasked interim report tables, the study was declared single-masked as planned in the protocol, with the participants, the site staff, and the investigator remaining masked. Study safety was evaluated by the GSK Safety Review Team using masked safety data. Additionally, a separate, project-independent internal GSK Safety Review Committee, composed of a study-independent GSK clinician, safety representative, statistician, and one external (non-GSK) clinical trials expert, reviewed safety at predefined timepoints based on unmasked safety results.

### Procedures

Enrolled participants received two intramuscular doses of either the unadjuvanted or adjuvanted Vi-CRM_197_+O:2-CRM_197_ vaccine 169 days (approximately 6 months) apart. A 6-month interval was chosen based on previous findings with glycoconjugate vaccines showing unsatisfactory boosting when a second dose was given shortly (1–2 months) after the priming dose (with no improvement by adding adjuvants), allowing sufficient time to observe a boosting effect and for B-memory cell maturity, while shorter intervals might hamper the boosting effect due to interference of antibodies induced by a first dose.

The Vi-CRM_197_+O:2-CRM_197_ low-dose and full-dose formulations contained 5 μg and 25 μg, respectively, of each Vi-CRM_197_ and O:2-CRM_197_ antigens. In adjuvanted formulations, both antigens were adsorbed on aluminium hydroxide (low-dose: 0·075 mg Al^3+^; full-dose: 0·375 mg Al^3+^). Dose escalation was done in three steps: step 1a, step 1b, and step 2. Progress into each step required a positive evaluation of safety data up to predefined timepoints from the previous steps by the internal GSK Safety Review Committee.

The low dose was obtained by fractional dosing of 0·1 mL of the full dose formulation. The monovalent, non-conjugated Vi capsular polysaccharide vaccine (Typhim Vi) and diphtheria toxoid-tetanus toxoid-acellular pertussis vaccine (Boostrix, GSK) were used as active controls for the first and the second vaccination, respectively. The Vi capsular polysaccharide vaccine was considered a more appropriate comparator than the oral Ty21a vaccine (both licensed in Europe), as it is a polysaccharide-based vaccine administered intramuscularly, has a similar presentation, and requires only one injection. The diphtheria toxoid-tetanus toxoid-acellular pertussis vaccine was chosen to maintain masking. Furthermore, safety profiles of both comparator vaccines are well characterised, and as they are not part of the routine vaccination schedule, they might provide additional benefit to the study participants. Details of the intervention administration are included in the appendix (p 7).

Six study visits were planned. Blood samples for safety assessment were collected at screening, day 1, day 8, day 169, and day 176, and for immunogenicity assessments at day 1, day 29, day 169, day 176, and day 197 (appendix p 6). Safety laboratory parameters are listed in the appendix (p 8).

Participants were followed up until day 337 (phone contact), approximately 6 months after dose 2, to evaluate the long-term safety through identification of potential serious adverse events (SAEs; appendix p 6).

### Outcomes

The primary study outcomes were the reactogenicity and safety profiles of the Vi-CRM_197_+O:2-CRM_197_ vaccine formulations. Solicited administration-site events (ie, pain, redness, and swelling) and systemic events (ie, fever, headache, myalgia, arthralgia, and fatigue) occurring within 7 days after vaccination were recorded by the participants in paper diaries. Adverse events definitions are included in the appendix (p 9). Safety laboratory (haematological and clinical chemistry) analyses were done before vaccination and 7 days after vaccination. Unsolicited adverse events were recorded until 28 days after vaccination. Pregnancies were documented from first vaccination until day 197. Any SAEs and adverse events or SAEs leading to withdrawal were collected until 28 days after dose 2 (day 197; primary outcome) and until day 337 (long-term safety follow-up; part of secondary outcome). These timepoints for adverse event collection are in line with the WHO guidelines on clinical evaluation of vaccines.^14^

Intensity of solicited events was graded on a scale of 0–3 (appendix p 10). The intensity of unsolicited adverse events (appendix p 11) and causality of unsolicited adverse events and SAEs were assessed by the investigator. The protocol toxicity grading scale for safety laboratory test results was based on the US Food and Drug Administration's Guidance for Industry (appendix p 12).^15^

The secondary outcomes included the long-term safety profile and the immunogenicity of the typhoid and paratyphoid A components of the vaccine formulations. Anti-Vi-specific and anti-O:2 IgG antibodies were measured by ELISA. Day 29 and day 197 (ie, 28 days after dose 1 and 2, respectively) were the main timepoints for immunogenicity assessment allowing sufficient time for the immune system to mount an immune response. Additionally, immunogenicity was assessed at day 176 (ie, 7 days after dose 2) to potentially capture an early booster response (ie, the rapid anamnestic or memory response).

The tertiary outcome included the immunogenicity profile of the paratyphoid A component of the vaccine formulations using a luminescence serum bactericidal assay. Additional tertiary outcomes, including the immunogenicity of the paratyphoid A vaccine component against heterologous *Salmonella* Paratyphi A strains with different O:2 antigen features, as measured by serum bactericidal assay, and antibody subclassing with the measurement of IgM and IgA against homologous *Salmonella* Paratyphi A strains, will be disclosed in a separate report. Immunological assays are described in the appendix (p 13). The full list of study objectives and outcomes is shown in the appendix (pp 14–15).

### Statistical analysis

96 participants were planned to be randomly assigned to achieve 82 evaluable participants. Since no confirmatory objectives were evaluated, this sample size was not driven by statistical considerations. The expected precision of the sample size for safety assessment was calculated based on the probability of detecting at least one adverse event considering different adverse events rates, while for immunogenicity assessment, on the number and percentage of participants with four-fold increase for the O:2 vaccine component (appendix p 16).

The reactogenicity and safety analyses were done on the solicited safety set, unsolicited safety set, and the exposed set. The primary immunogenicity analysis was done on the per-protocol set defined by timepoint; a secondary analysis based on the full analysis set for immunogenicity, defined by timepoint, was done to complement the per-protocol set analysis. The analysis sets are described in the appendix (pp 16–17).

The percentages of participants reporting solicited events were tabulated with exact 95% CIs. The percentages of participants with unsolicited adverse events with exact 95% CIs were tabulated by Medical Dictionary for Regulatory Activities System Organ Class and Preferred Terms.

Geometric mean concentrations (GMCs) and within-participant geometric mean ratios (GMRs) of anti-Vi IgG and anti-O:2 IgG antibodies with 95% CIs, also reported as geometric mean fold-rise (GMFR), were defined against the baseline value (appendix p 17).

Seroresponse rates, calculated with their exact 95% CIs, were defined as: (1) the number and percentage of participants with anti-Vi IgG antibody concentrations ≥4·3 μg/mL, a threshold defined as sustained protection,^16^ and ≥2·0 μg/mL, estimated to predict short-term protection in a paediatric study^17^ (these thresholds were used for licensure of typhoid conjugate vaccines); and (2) the number and percentage of participants with ≥4-fold increase in anti-O:2 IgG antibody concentrations 28 days after vaccination compared with baseline (day 1). Geometric mean titres (GMTs), within-participant GMRs, and percentages of participants with ≥4-fold increase in serum bactericidal activity against *Salmonella* Paratyphi A were calculated with their 95% CIs.

A general linear model with treatment as covariate was used to perform unadjusted pairwise comparison. A linear mixed model with repeated measures with fixed effects for treatment, timepoint, interaction between treatment and timepoint, and baseline immunogenicity value as covariate, and a repeated timepoint effect within a participant under an exchangeable covariance matrix, was used to perform adjusted pairwise comparison between adjuvanted versus unadjuvanted formulations after each dose. Variances between the intervention groups were not considered equal unless the assumption led to convergence issues. Restricted maximum likelihood was used to estimate the variance components and Satterthwaite's approximation was used to calculate the degrees of freedom for the tests. Given the descriptive nature of this trial, all testing was exploratory and no adjustment for multiplicity was applied. All statistical analyses were done with SAS version 9.4.

### Role of the funding source

The funder of the study (Wellcome Trust) was not involved in study design, data collection, data analysis, data interpretation, or writing of the report. This publication was sponsored by GSK. GSK was involved in all stages of the study conduct and analysis and took responsibility for all costs associated with the development and the publishing of this report.

## Results

**Figure 1:**
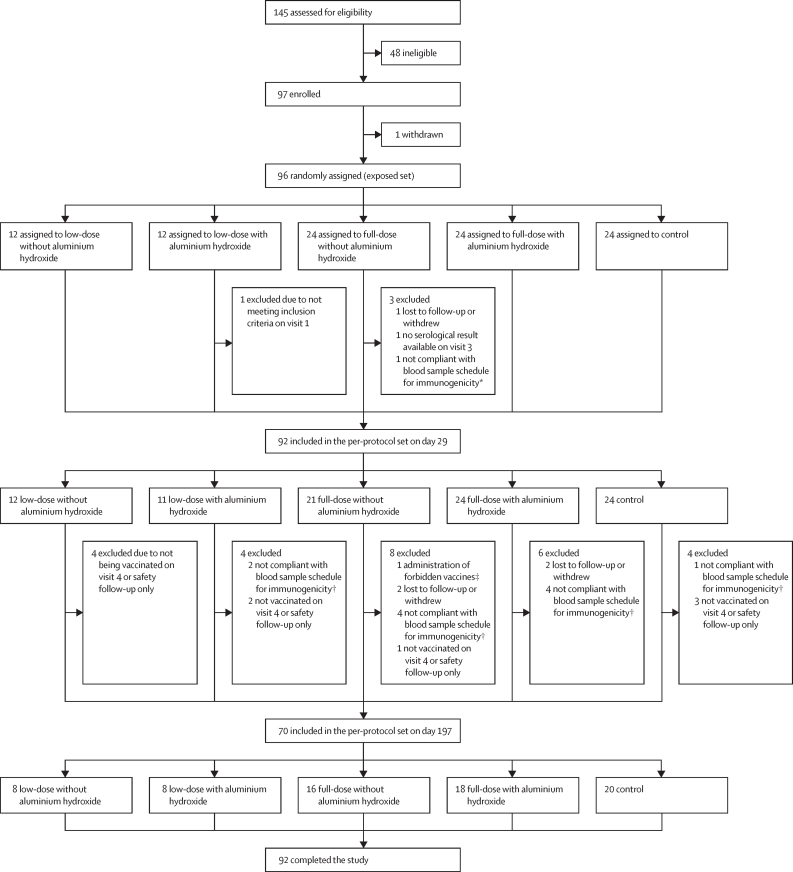
Trial profile *Out of interval between visit 1 and visit 3. †Out of interval between visit 4 and visit 6. ‡Administration of concomitant vaccines forbidden in the protocol between visit 5 and visit 6.

Participants were screened between Nov 28, 2022, and May 11, 2023. Of the 145 participants assessed for eligibility, 96 were randomly assigned and vaccinated (the exposed set) to low-dose without aluminium hydroxide (n=12), low-dose with aluminium hydroxide (n=12), full-dose without aluminium hydroxide (n=24), full-dose with aluminium hydroxide (n=24), and control (n=24). Four participants were withdrawn and 92 completed the study (figure 1; appendix p 18); the last participant completed the study on April 2, 2024. The mean age was 29·2 years; 66 (69%) of 96 participants were women (table 1). Demographic characteristics of participants in the per-protocol set are in the appendix (p 19). Protocol deviations leading to the exclusion of individuals from any analyses and from the per-protocol set are described in figure 1 and the appendix (pp 18, 20–21).

**Table 1:** Demographic and baseline characteristics of the study participants (exposed set)

		**Low-dose without aluminium hydroxide group (N=12)**	**Low-dose with aluminium hydroxide group (N=12)**	**Full-dose without aluminium hydroxide group (N=24)**	**Full-dose with aluminium hydroxide group (N=24)**	**Control group (N=24)**	**Total (N=96)**
Age, years		31·9 (10·8)	31·8 (8·4)	27·0 (9·2)	27·1 (9·8)	30·7 (10·6)	29·2 (9·9)
Sex							
	Female	11 (92%)	6 (50%)	14 (58%)	17 (71%)	18 (75%)	66 (69%)
	Male	1 (8%)	6 (50%)	10 (42%)	7 (29%)	6 (25%)	30 (31%)
Height, cm		168·6 (3·9)	174·3 (10·7)	173·5 (10·4)	173·7 (7·7)	169·5 (6·5)	172·0 (8·4)
Weight, kg		68·7 (10·3)	79·3 (13·1)	76·0 (13·3)	71·3 (13·2)	71·4 (13·3)	73·2 (13·1)
BMI, kg/m2		24·2 (3·6)	26·3 (4·7)	25·2 (3·7)	23·5 (3·4)	24·8 (4·5)	24·7 (4·0)

Data are mean (SD) or n (%). N=number of participants with at least one administered dose. n (%)=number (percentage) of participants in each category.

**Figure 2:**
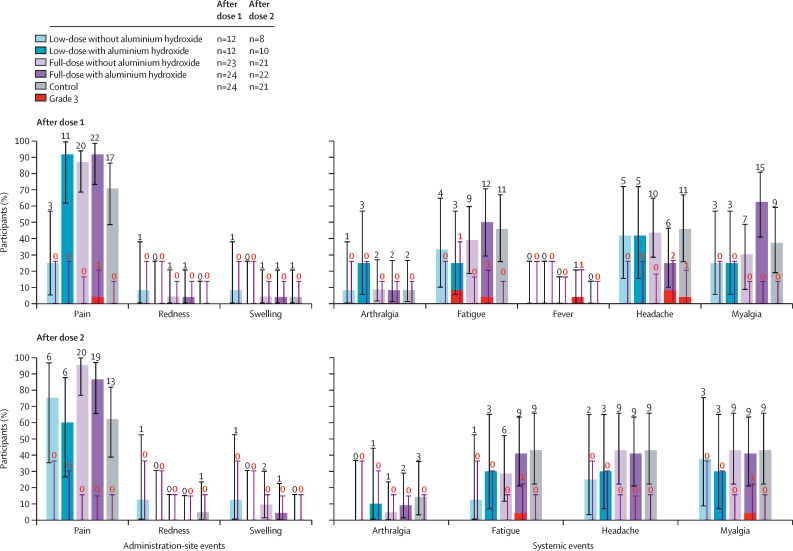
Incidence of solicited administration-site and systemic events, solicited safety set The error bars represent 95% CIs. The numbers above the error bars represent the number of participants per group reporting events of any grade and grade 3. n=number of participants with available results.

The incidence of solicited events ranged from nine (75%) of 12 participants (95% CI 42·8–94·5) in the low-dose without aluminium hydroxide group to all 24 (100%) participants (85·8–100) in the full-dose with aluminium hydroxide group; grade 3 solicited events were reported by one (8%) of 12 participants (0·2–38·5) in the low-dose with aluminium hydroxide group and four (17%) of 24 (4·7–37·4) in the full-dose with aluminium hydroxide group, versus one (4%) of 24 (0·1–21·1) in the control group (table 2; appendix p 22). The incidence of solicited administration-site events ranged from six (50%) of 12 participants (21·1–78·9) in the low-dose without aluminium hydroxide group to 23 (96%) of 24 (78·9–99·9) in the full-dose with aluminium hydroxide group, versus 22 (92%) of 24 (73·0–99·0) in the control group; the most common solicited administration-site event in all groups was pain (figure 2; appendix p 23). Grade 3 pain was reported after dose 1 by one (4%) of 24 participants (0·1–21·1) in the full-dose with aluminium hydroxide group (figure 2). The incidence of solicited systemic events ranged from eight (67%) of 12 participants (34·9–90·1) in the low-dose with and without aluminium hydroxide to 20 (83%) of 24 (62·6–95·3) in the full-dose with aluminium hydroxide, versus 21 (88%) of 24 (67·6–97·3) in the control group; the most common solicited systemic events across all groups were fatigue, headache, and myalgia (figure 2; appendix p 23). Grade 3 solicited systemic events were reported in the adjuvanted groups and the control group (figure 2; appendix p 22).

**Table 2:** Summary of safety results: solicited events (solicited safety set) and unsolicited adverse events (unsolicited safety set) reported during the 7-day and 28-day post-vaccination period, respectively, overall per participant after any dose

		**Low-dose without aluminium hydroxide group**	**Low-dose with aluminium hydroxide group**	**Full-dose without aluminium hydroxide group**	**Full-dose with aluminium hydroxide group**	**Control group**
**Solicited events**						
Any event		9/12 (75·0%); 42·8–94·5	12/12 (100%); 73·5–100	22/23 (95·7%); 78·1–99·9	24/24 (100%); 85·8–100	22/24 (91·7%); 73·0–99·0
	Grade 3	0/12 (0%); 0·0–26·5	1/12 (8·3%); 0·2–38·5	0/23 (0%); 0·0–14·8	4/24 (16·7%); 4·7–37·4	1/24 (4·2%); 0·1–21·1
Administration-site events		6/12 (50·0%); 21·1–78·9	11/12 (91·7%); 61·5–99·8	21/23 (91·3%); 72·0–98·9	23/24 (95·8%); 78·9–99·9	22/24 (91·7%); 73·0–99·0
	Grade 3	0/12 (0%); 0·0–26·5	0/12 (0%); 0·0–26·5	0/23 (0%); 0·0–14·8	1/24 (4·2%); 0·1–21·1	0/24 (0%); 0·0–14·2
Systemic events		8/12 (66·7%); 34·9–90·1	8/12 (66·7%); 34·9–90·1	19/23 (82·6%); 61·2–95·0	20/24 (83·3%); 62·6–95·3	21/24 (87·5%); 67·6–97·3
	Grade 3	0/12 (0%); 0·0–26·5	1/12 (8·3%); 0·2–38·5	0/23 (0%); 0·0–14·8	4/24 (16·7%); 4·7–37·4	1/24 (4·2%); 0·1–21·1
**Unsolicited adverse events**						
Any event		7/12 (58·3%); 27·7–84·8	10/12 (83·3%); 51·6–97·9	19/24 (79·2%); 57·8–92·9	16/24 (66·7%); 44·7–84·4	14/24 (58·3%); 36·6–77·9
	Grade 3	0/12 (0%); 0·0–26·5	1/12 (8·3%); 0·2–38·5	1/24 (4·2%); 0·1–21·1	3/24 (12·5%); 2·7–32·4	1/24 (4·2%); 0·1–21·1
Related		3/12 (25·0%); 5·5–57·2	5/12 (41·7%); 15·2–72·3	10/24 (41·7%); 22·1–63·4	9/24 (37·5%); 18·8–59·4	6/24 (25·0%); 9·8–46·7
	Grade 3-related	0/12 (0%); 0·0–26·5	1/12 (8·3%); 0·2–38·5	1/24 (4·2%); 0·1–21·1	2/24 (8·3%); 1·0–27·0	0/24 (0%); 0·0–14·2

Data are n/N (%); 95% CI. N=number of participants with at least one administered dose in the solicited or unsolicited safety set. n (%)=number (percentage) of participants reporting an event at least once.

All solicited administration-site events resolved within 7 days after vaccination except for events reported in two participants for whom the events ongoing 7 days after vaccination were redness and swelling, all non-medically attended and classified as grade 0 according to the protocol grading scale (all were ≤9 mm). One participant (low-dose with aluminium hydroxide) reported redness and swelling after dose 1 (resolved within 12 days), and after dose 2 (resolved within 30 days); another participant (full-dose with aluminium hydroxide) reported redness after dose 1 (resolved within 10 days)*.*

The incidence of unsolicited adverse events ranged from seven (58%) of 12 participants (95% CI 27·7–84·8) in the low-dose without aluminium hydroxide to ten (83%) of 12 (51·6–97·9) in the low-dose with aluminium hydroxide group, versus 14 (58%) of 24 (36·6–77·9) in the control group (table 2; appendix p 24). The most common (≥12·5% of participants in either of the treatment groups) unsolicited adverse events were nasopharyngitis (ranging from three [13%] of 24 [2·7–32·4] in the full-dose without aluminium hydroxide group to four [33%] of 12 [9·9–65·1] in the low-dose with aluminium hydroxide group), headache (control: four [17%] of 24 [4·7–37·4]), contusion (full-dose without aluminium hydroxide: three [13%] of 24 [2·7–32·4]), and musculoskeletal stiffness (full-dose without aluminium hydroxide: three [13%] of 24 [2·7–32·4]). Six participants had grade 3 unsolicited adverse events after dose 1: one (4%) of 24 (0·1–21·1) in the full-dose without aluminium hydroxide group and the control group, one (8%) of 12 (0·2–38·5) in the low-dose with aluminium hydroxide group, and three (13%) of 24 (2·7–32·4) in the full-dose with aluminium hydroxide group; no grade 3 unsolicited adverse events were reported after dose 2. The incidence of unsolicited adverse events considered related to vaccination ranged from three (25%) of 12 participants (5·5–57·2) in the low-dose without aluminium hydroxide group and six (25%) of 24 (9·8–46·7) in the control group to ten (42%) of 24 (22·1–63·4) in the full-dose without aluminium hydroxide group (table 2; appendix p 24). Further details on unsolicited adverse events are in the appendix (p 25). Four grade 3 unsolicited adverse events considered related to vaccination were reported: a case of migraine (one [8%] of 12 participants [0·2–38·5] in the low-dose with aluminium hydroxide group), and three events of clinically relevant decreased haemoglobin levels (one [4%] of 24 participants [0·1–21·1] in the full-dose without aluminium hydroxide group and two [8%] of 24 participants [1·0–27·0] in the full-dose with aluminium hydroxide group; table 2; appendix p 25). The events of decreased haemoglobin levels (change from baseline) were classified as grade 3 according to the protocol toxicity grade but remained within local laboratory normal ranges; for the participant from the full-dose without aluminium hydroxide group, the event (reported 8 days after dose 1) led to withholding of further study intervention, resolved without medication and the participant completed the applicable follow-up in the study. Two other participants had adverse events considered not related to vaccination, for which further study intervention administrations were also withheld (appendix p 25). No SAEs and no pregnancies were reported in the study. Safety laboratory analyses did not reveal specific trends or concerns; most values remained within normal ranges or were of grade 1 intensity, and all were deemed clinically not significant by the investigator.

**Figure 3:**
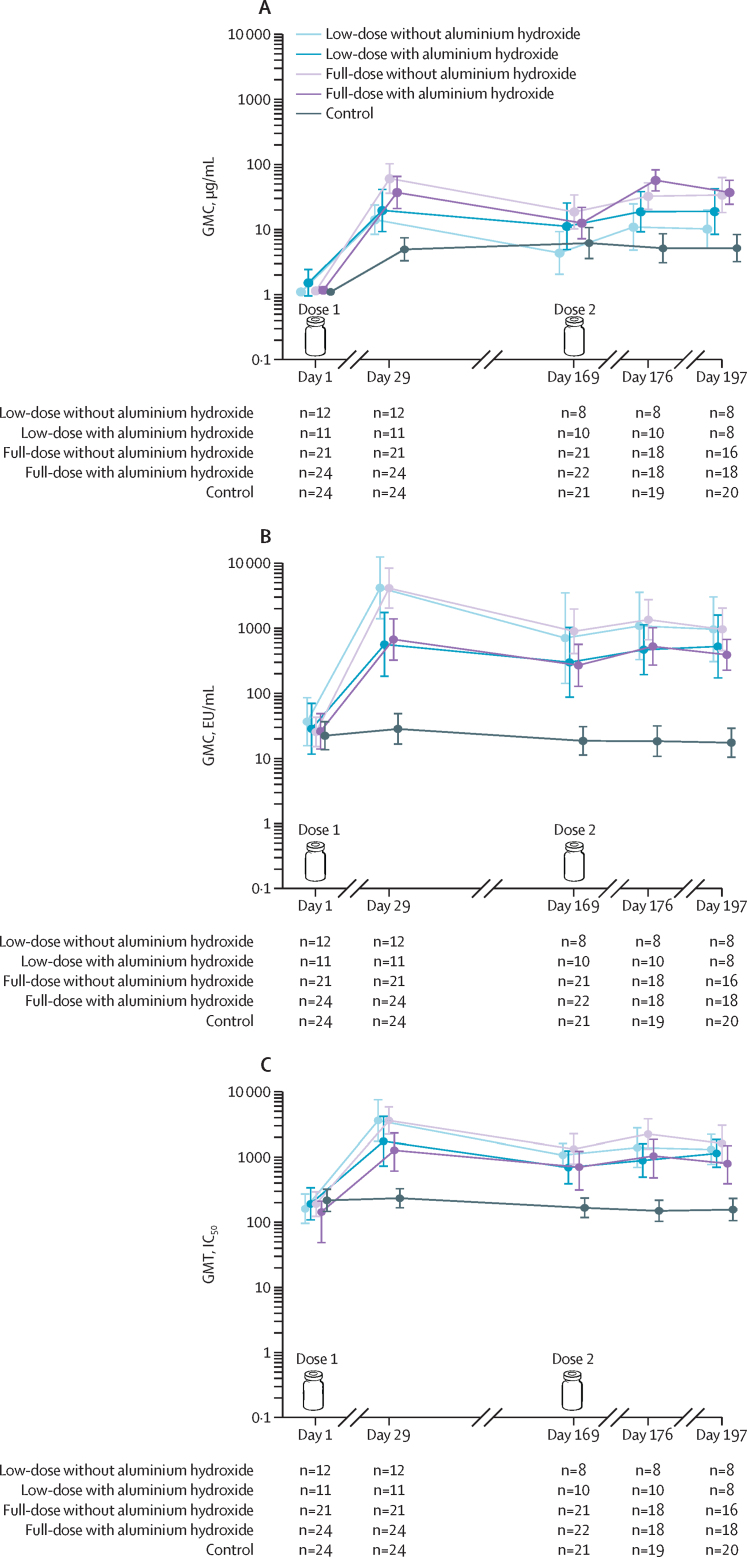
Immune responses to vaccine antigens, per-protocol set (A) Anti-Vi IgG. (B) Anti-O:2 IgG GMCs (by ELISA). (C) GMTs for bactericidal antibodies against *Salmonella* Paratyphi A (by serum bactericidal assay). The error bars represent 95% CIs. The vials represent vaccination doses 1 and 2. The per-protocol set was defined by timepoint. EU=ELISA units. GMC=geometric mean concentration. GMT=geometric mean titre. IC_50_=50% inhibition of bacterial growth. n=number of participants with available results per group.

In the per-protocol set, anti-Vi IgG GMCs increased at 1 month after dose 1 (day 29) in all vaccine formulation groups and remained at similar levels and well above the baseline until before dose 2 (day 169); the second dose did not show a boosting effect as evidenced by similar GMCs on day 197 versus day 29 (figure 3A). Overall, full-dose without aluminium hydroxide and full-dose with aluminium hydroxide formulations seemed to induce the highest anti-Vi IgG GMFR at day 29 (53·01-fold [95% CI 31·94–87·99] and 31·55-fold [18·74–53·11], respectively) and day 197 (29·22-fold [15·71–54·35] and 31·04-fold [23·14–41·65], respectively) over pre-vaccination values (day 1). No substantial increase in GMCs was observed in the control group (GMR at day 29: 4·50 [2·93–6·90]; at day 197: 4·70 [2·85–7·74]; appendix p 26). Reverse cumulative distribution curves of anti-Vi IgG antibody concentrations are in the appendix (p 27). 1 month after dose 1, among participants seronegative at baseline (ie, with anti-Vi IgG antibody concentrations <4·3 μg/mL), eight (89%) of nine participants (51·8–99·7) in the low-dose with aluminium hydroxide group, 22 (96%) of 23 (78·1–99·9) in the full-dose with aluminium hydroxide group, 12 (100%) of 12 (73·5–100) in the low-dose without aluminium hydroxide group, and 21 (100%) of 21 (83·9–100) in the full-dose without aluminium hydroxide group reached anti-Vi IgG concentrations ≥4·3 μg/mL, versus 13 (54%) of 24 (32·8–74·4) in the control group (Vi capsular polysaccharide vaccine). 1 month after dose 2 (day 197), these proportions were five (83%) of six participants (35·9–99·6) in the low-dose with aluminium hydroxide group, seven (88%) of eight (47·3–99·7) in the low-dose without aluminium hydroxide group, 15 (94%) of 16 (69·8–99·8) in the full-dose without aluminium hydroxide group, and 17 (100%) of 17 (80·5–100) in the full-dose with aluminium hydroxide group, versus 12 (60%) of 20 (36·1–80·9) in the control group. Numbers and percentages of participants with anti-Vi IgG concentrations ≥2·0 μg/mL are reported in the appendix (p 26). Pairwise comparisons of the adjusted and unadjusted GMRs for anti-Vi IgG between full-dose with and without aluminium hydroxide and between low-dose with and without aluminium hydroxide did not indicate any difference (appendix pp 28–29).

In the per-protocol set, anti-O:2 IgG GMCs increased 1 month after dose 1 in all vaccine formulation groups and remained at similar levels and well above the baseline until before dose 2; the second dose did not show a boosting effect as evidenced by similar GMCs on day 197 versus day 29 (figure 3B). Low-dose without aluminium hydroxide and full-dose without aluminium hydroxide groups induced the highest anti-O:2 IgG GMFR at day 29 (114·19-fold [95% CI 44·83–290·86] and 162·61-fold [91·17–290·04], respectively) and day 197 (35·66-fold [10·36–122·82] and 51·16-fold [27·32–95·79], respectively) over pre-vaccination. No response was observed in the control group (GMR at day 29: 1·27 [1·02–1·60]; day 197: 0·95 [0·81–1·12]; appendix p 30). Reverse cumulative distribution curves of anti-O:2 IgG antibody concentrations are shown in the appendix (p 31). 1 month after dose 1, nine (82%) of 11 participants (48·2–97·7) in the low-dose with aluminium hydroxide group, 20 (83%) of 24 (62·6–95·3) in the full-dose with aluminium hydroxide group, 12 (100%) of 12 (73·5–100) in the low-dose without aluminium hydroxide group, and 21 (100%) of 21 (83·9–100) in the full-dose without aluminium hydroxide group, versus one (4%) of 24 (0·1–21·1) in the control group, achieved at least four-fold increase from baseline in anti-O:2 IgG levels. 1 month after dose 2, these proportions were seven (88%) of eight participants (47·3–99·7), 16 (89%) of 18 (65·3–98·6), eight (100%) of eight (63·1–100), and 15 (94%) of 16 (69·8–99·8), versus none (0%) of 20 (0·0–16·8), respectively (appendix p 30). A pairwise comparison of the unadjusted and adjusted GMRs for anti-O:2 IgG suggested that full-dose without aluminium hydroxide was more immunogenic than full-dose with aluminium hydroxide (unadjusted GMR: 0·16; 0·06–0·43 [p=0·0003] after dose 1; 0·41; 0·17–0·97 [p=0·043] after dose 2); comparison of unadjusted GMRs after dose 1 suggested that low-dose without aluminium hydroxide was more immunogenic than low-dose with aluminium hydroxide (unadjusted GMR: 0·13; 0·04–0·52 [p=0·0040]; appendix pp 28–29).

In the per-protocol set, 1 month after dose 1, GMTs for bactericidal antibodies against *Salmonella* Paratyphi A increased substantially in all vaccine formulation groups and remained above the baseline levels until before dose 2. The second dose did not show a boosting effect as evidenced by similar GMTs at day 197 versus day 29 (figure 3C). After dose 1, the low-dose and full-dose unadjuvanted formulations induced the highest GMFR in *Salmonella* Paratyphi A bactericidal antibody titres over pre-vaccination value at day 1 (22·45-fold [95% CI 8·22–61·27] and 19·08-fold [10·61–34·32], respectively). 1 month after dose 2, the full-dose without aluminium hydroxide formulation induced the highest GMFR (11·08-fold [6·14–20·02]) over pre-vaccination. No response was observed in the control group (GMR at day 29: 1·08 [0·90–1·30]; at day 197: 0·82 [0·61–1·12]) (appendix p 32). Reverse cumulative distribution curves of bactericidal antibody titres against *Salmonella* Paratyphi A are shown in the appendix (p 33). 1 month after dose 1, eight (73%) of 11 participants (39·0–94·0) in the low-dose with aluminium hydroxide group, 18 (75%) of 24 (53·3–90·2) in the full-dose with aluminium hydroxide group, 19 (91%) of 21 (69·6–98·8) in the full-dose without aluminium hydroxide group, and 11 (92%) of 12 (61·5–99·8) in the low-dose without aluminium hydroxide group had at least a four-fold increase from baseline in bactericidal antibody titres against *Salmonella* Paratyphi A. 1 month after dose 2, these proportions were six (75%) of eight participants (34·9–96·8), 13 (72%) of 18 (46·5–90·3), 14 (88%) of 16 (61·7–98·4), and four (50%) of eight (15·7–84·3), respectively; no participants in the control group had a four-fold increase from baseline (appendix p 32). A pairwise comparison of unadjusted and adjusted GMRs for bactericidal antibody titres against *Salmonella* Paratyphi A after dose 1 showed that full-dose without aluminium hydroxide had a higher bactericidal activity than full-dose with aluminium hydroxide (unadjusted GMR: 0·35; 0·17–0·72 [p=0·0046]; adjusted GMR: 0·43; 0·22–0·84 [p=0·014]); a similar trend was observed after dose 2 (unadjusted GMR: 0·49; 0·24–1·02 [p=0·057]; appendix pp 28–29). Results from the full analysis set were in line with the analyses based on the per-protocol set and are shown in the appendix (pp 34–36). A visual representation of the study key findings is shown in the appendix (p 37).

## Discussion

In this phase 1 study, no safety concerns precluding further vaccine development were observed for all investigational Vi-CRM_197_+O:2-CRM_197_ formulations. The first dose of all vaccine formulations induced robust immune responses to both Vi and O:2 antigens in healthy European adults, without boosting effect of the second dose.

The incidence of solicited administration-site events was lower in the low-dose without aluminium hydroxide group compared with other groups likely due to the fractional administration for the low-dose groups which resulted in lower volume administered (0·1 mL compared with 0·5 mL), and the effect of the aluminium hydroxide adjuvant when comparing with the low-dose with aluminium hydroxide formulation. In general, aluminium hydroxide-adjuvanted vaccines are associated with higher incidence of pain after vaccination and redness compared with non-adjuvanted vaccines.^18^ In this study, pain was the most frequent solicited administration-site symptom, similarly to a recent phase 1 study among recipients of another bivalent paratyphoid A-typhoid conjugate candidate vaccine (Sii-PTCV),^19^ and is commonly observed following administration of other typhoid conjugate vaccines, including the licensed typhoid conjugate vaccines and the WHO-prequalified monovalent Vi-CRM_197_ vaccine.^16^, ^20^, ^21^, ^22^, ^23^, ^24^ Fatigue, headache, and myalgia were the most common solicited systemic events, consistent with safety data obtained for the Vi-CRM_197_ vaccine,^24^ the Vi polysaccharide-diphtheria toxoid and Vi polysaccharide-tetanus toxoid vaccines,^20^, ^25^ and the bivalent paratyphoid A-typhoid conjugate vaccine (Sii-PTCV) vaccine.^19^

A robust immune response in terms of anti-Vi and anti-O:2 IgG antibody concentrations, and serum bactericidal assay titres against *Salmonella* Paratyphi A was observed 1 month after dose 1 for all formulations, compared with the control vaccine (Vi capsular polysaccharide vaccine). Similarly, studies with the Vi polysaccharide-tetanus toxoid vaccines and Vi polysaccharide-diphtheria toxoid vaccines reported higher anti-Vi responses with typhoid conjugate vaccines compared with Vi capsular polysaccharide vaccines.^26^, ^27^ This was expected because conjugate vaccines generally induce much higher antibody levels and a T-dependent immune response compared with non-conjugated polysaccharide vaccines such as Vi capsular polysaccharide vaccines.^26^, ^28^ Anti-Vi seroresponse rates, using the threshold for anti-Vi IgG of ≥4·3 μg/mL, defined as criterion of sustained protection for at least 4 years during licensing of the conjugate monovalent Vi-CRM_197_ vaccine in India,^16^ were substantially higher across all Vi-CRM_197_+O:2-CRM_197_ formulations (≥89% 1 month after dose 1) compared with the control group who received Vi capsular polysaccharide vaccine (54%). Similarly high responses were observed with a single dose of the licensed Vi-CRM_197_ vaccine,^16^, ^29^, ^30^ and with the Sii-PTCV vaccine.^19^

The unadjuvanted formulations induced higher anti-O:2 IgG and serum bactericidal assay responses than the adjuvanted formulations at all timepoints. 1 month after dose 1, high GMFRs were observed for anti-O:2 IgG and serum bactericidal assay titres (ELISA: low-dose: 114·19; full-dose: 162·61; serum bactericidal assay: 22·45; 19·08, respectively), similar to the Sii-PTCV vaccine (ELISA: 80·02; serum bactericidal assay: 19·40).^19^ However, considering differences between our and the previous study in terms of the vaccine construct (different carrier protein), population, setting (European versus endemic population), and ELISA and serum bactericidal assays used, direct immunogenicity comparisons might not be appropriate.

The persistence of the immune response for both Vi and O:2 vaccine components up to 6 months after dose 1, and the lack of boosting effect of the second dose need to be considered when selecting the vaccine schedule. Similar findings were reported in a study with a candidate conjugate O-specific polysaccharide-tetanus toxoid vaccine administered 6 weeks apart to children aged 2–4 years, where a significant rise in anti-lipopolysaccharide IgG levels was induced by the first injection with no booster effect of the second injection.^31^

Overall, a higher anti-O:2 IgG response and a trend for a higher anti-Vi IgG response were observed for the full-dose without aluminium hydroxide compared with the full-dose with aluminium hydroxide formulation. Based on the pairwise comparisons of the unadjusted GMRs, we did not find beneficial effects of the aluminium hydroxide adjuvant on anti-Vi IgG responses compared with non-aluminium hydroxide formulations. Additionally, comparisons of both unadjusted and adjusted GMRs for anti-O:2 suggested that the full-dose without aluminium hydroxide was more immunogenic than full-dose with aluminium hydroxide, as measured by both ELISA and serum bactericidal assay. The lower immunogenicity observed for aluminium hydroxide-adjuvanted formulations might result from reduced stability of the conjugate when adsorbed on aluminium hydroxide, potentially affecting the conjugate's integrity.^32^

Although there is no immune correlate of protection for paratyphoid A, showing the functionality of antibodies generated through vaccination, and thus their ability to kill *Salmonella* Paratyphi A strains in vitro, might indicate potential protective effect of the vaccine.^33^ Serum bactericidal assay was also used to test clinical sera in the recent phase 1 trial with the Sii-PTCV vaccine.^19^ Notably, serum bactericidal activity has been recognised as a correlate of protection for other Gram-negative bacteria (eg, meningococcus),^34^ and the serum bactericidal assay is generally considered a prioritised assay in *Salmonella* research. Furthermore, previous studies showed a possible correlation of the anti-Vi IgA concentrations with protection against typhoid fever, suggesting that anti-Vi IgA might serve as a surrogate marker predicting protection from disease following vaccination.^35^, ^36^ In addition to IgA, elevated levels of Vi-specific IgG and IgM were also found in protected individuals vaccinated with Vi capsular polysaccharide vaccines.^36^ Thus, additional analyses are planned to evaluate the ability of O:2-CRM_197_ to induce antigen-specific IgA, and the potential correlation of antigen-specific IgA and IgM antibody levels with their bactericidal activity against a panel of *Salmonella* Paratyphi A strains. Altogether, the immunogenicity results indicate that all Vi-CRM_197_+O:2-CRM_197_ formulations induced a high immune response after dose 1.

The main strengths of this study include multiple formulations tested with low and full antigen doses with or without the aluminium hydroxide adjuvant, and the use of the licensed vaccines, including the comparator typhoid vaccine, and the diphtheria toxoid-tetanus toxoid-acellular pertussis vaccine, providing additional benefits to the control group from the study participation. Additionally, an extended 6-month safety follow-up after dose 2 allowed to collect potential late-occurring SAEs.

A potential limitation of this study is the absence of single-dose groups to assess immunogenicity of a single dose over a longer period. Additionally, the comparator vaccine was not a typhoid conjugate vaccine; however, Vi capsular polysaccharide vaccine is the only typhoid vaccine licensed in Europe and its safety profile is well characterised, supporting its use in this study whose primary objective was safety assessment. Furthermore, it is not known whether in the absence of an established correlate of protection, the anti-O:2 antibody levels observed in this study are indicative of vaccine efficacy. Finally, no statistical corrections were applied for the pairwise comparisons of the GMRs to adjust for multiplicity, potentially increasing the risk of Type I error; however, such analysis was not planned in the protocol, given this was a secondary objective, and considering the nature of the study (phase 1).

In conclusion, no safety concerns with the bivalent conjugate Vi-CRM_197_+O:2-CRM_197_ candidate vaccine were identified when administered with or without the adjuvant. A single dose of all vaccine formulations was immunogenic, with the full-dose without aluminium hydroxide formulation showing an overall better immunogenicity. These results support further clinical development of the vaccine for the prevention of both typhoid and paratyphoid enteric fever in infants and older age groups in endemic countries, including studies with a direct comparison with a licensed typhoid conjugate vaccine and in target populations. A single dose of the full-dose without aluminium hydroxide vaccine formulation is being assessed in a phase 1 study in healthy adults in an endemic country (India; Clinical Trials Registry India code CTRI/2025/07/090155); a phase 2 study is planned in target populations.

**This online publication has been corrected. The corrected version first appeared at thelancet.com/infection on February 4, 2026**

### Contributors

### Data sharing

Please refer to the GSK weblink to access GSK's data sharing policies and, as applicable, seek anonymised subject-level data via the link https://www.gsk-studyregister.com/en/. Clicking on Learn More under the Sharing Clinical Trial Data section will redirect you to another page, GSK – Vivli, which contains the information about data sharing. As per GSK policy, data will be shared once the product is approved by regulators or development is terminated across all indications for investigational medicines or vaccines.

## Declaration of interests

IDC acts as principal investigator for vaccine trials conducted on behalf of the University of Antwerp, for which the University obtains research grants from vaccine manufacturers. IDC received no personal remuneration for this work. IDC is also a member of a data safety monitoring board for International Vaccine Institute. MAG, ES, CF, EM, RLG, SN, MC, LM, VC, OR, GLC, AS-B, PT, FM, FBS, SR, and AKA are employed by GSK. UNN was an employee of GSK at the time the study was conducted and is currently an employee of the Gates Foundation. MAG, EM, RLG, MC, VC, OR, GLC, AS-B, FM, FBS, SR, UNN, and AKA hold financial equities in GSK. EM declares receiving support for attending meetings or travel from GSK. PVD obtains grants and research agreements for the conduct of vaccine trials from GSK and other vaccine manufacturers through his institution, the University of Antwerp. PVD received no personal remuneration for this work. PVD is also a member of a data safety monitoring board for Janssen Vaccines. FM received funds from the Wellcome Trust. FBS received grants or contracts from the Gates Foundation. SR received grants from the Wellcome Trust (via Novartis Vaccines Institute for Global Health [now GSK Vaccines Institute for Global Health]) and declares being a co-applicant for the patent of the vaccine formulation N425315GB. AKA received grants or contracts for the study funding through the Wellcome Trust and GSK, and support for attending meetings or travel via funding from GSK. KW declares no competing interests. All authors declare no non-financial relationships and activities.
