# Correction to *Lancet Infect Dis* 2026; published online Jan 23. https://doi.org/10.1016/S1473-3099(25)00730-3

**DOI:** 10.1016/S1473-3099(26)00066-6

**Published:** 2026-04

*De Coster I, AbdelGhany M, Sarakinou E, et al. Safety and immunogenicity of a conjugate vaccine candidate against* Salmonella enterica *serovars Typhi and Paratyphi A in healthy adults in Europe: a phase 1 randomised controlled trial.* Lancet Infect Dis *2026; published online Jan 23. https://doi.org/10.1016/S1473-3099(25)00730-3*—In the Acknowledgments of this Article, the funding statement should have been “This work was funded by the Wellcome Trust [089102/Z/09/Z] and this publication was sponsored by GSK.” This correction has been made to the online version as of Feb 4, 2026, and will be made to the printed version.

