# Supplementary material for: Safety and immunogenicity of a conjugate vaccine candidate against Salmonella enterica serovars Typhi and Paratyphi A in healthy adults in Europe: a phase 1 randomised controlled trial
Source: Lancet Infect Dis. 2026 Jun;26(6):638–50. doi: 10.1016/S1473-3099(25)00730-3 (PMC13215975; doi:10.1016/S1473-3099(25)00730-3)
Supplement: Supplementary appendix [file mmc1.pdf]

THE LANCET
Infectious Diseases

Supplementary appendix

This appendix formed part of the original submission and has been peer reviewed. We post it as supplied by the authors.

Supplement to: Louie T, Ribble W, Boccumini L, et al. Safety and efficacy of CRS3123 in adults with a primary episode or first recurrence of *Clostridioides difficile* infection: a phase 2, randomised, double-blind, multicentre, vancomycin-controlled study. *Lancet Infect Dis* 2026; published online Jan 22. https://doi.org/10.1016/S1473-3099(25)00721-2.

Supplementary appendix

Table of Contents

Plain language summary 2
Supplementary methods 3
Inclusion criteria 3
Exclusion criteria 3
Supplementary table S1 5
Supplementary figure S1 6
Details of intervention administration 7
Supplementary table S2 8
Definitions of adverse events 9
Supplementary table S3 10
Supplementary table S4 11
Supplementary table S5 12
Details of immunological assays 13
Supplementary table S6 14
Details of statistical analyses 16
Supplementary results 18
Supplementary figure S2 18
Supplementary table S7 19
Summary of protocol deviations leading to elimination from any analyses..20
Supplementary table S8 21
Supplementary safety results 22
Supplementary figure S3..22
Details of solicited events 23
Supplementary figure S4..24
Details of unsolicited AEs 25
Supplementary immunogenicity results 26
Supplementary table S9 26
Supplementary figure S5..27
Supplementary table S10 28
Supplementary table S11 30
Supplementary figure S6..31
Supplementary table S12 32
Supplementary figure S7..33
Supplementary table S13 34
Supplementary table S14 35
Supplementary table S15 36
Supplementary figure S8..37
Supplementary references 38

Plain language summary

What is the context?

- Enteric fever is a serious and potentially fatal disease caused by infection with bacteria called *Salmonella* Typhi and *Salmonella* Paratyphi A. Both are transmitted via food or water contaminated with feces of an infected person, or via direct person-to-person contact.
- Enteric fever affects the whole body, including the intestines and the bloodstream. Symptoms and signs include fever, diarrhea, tiredness, enlarged spleen and liver, and a typical skin rash.
- The majority of enteric fever cases occur in children of school age or younger, who live in areas with frequent *Salmonella* infections such as Africa and South Asia.
- *Salmonella* Typhi causes most cases of enteric fever, but the proportion of cases caused by *Salmonella* Paratyphi A, relative to *Salmonella* Typhi, has been increasing in recent years.
- Treatment of enteric fever is challenging due to *Salmonella* strains that are increasingly resistant to many antibiotics.
- Several vaccines against *Salmonella* Typhi exist, but no vaccine against *Salmonella* Paratyphi A is currently available.

What is new?

- The candidate vaccine, Vi-CRM$_{197}$+O:2-CRM$_{197}$, contains antigens of both *Salmonella* Typhi (Vi antigen) and *Salmonella* Paratyphi A (O:2 antigen). Four different formulations of the vaccine were generated, combining different antigen levels (low or full dose) with or without an aluminium-based adjuvant (a substance used to improve the ability to induce an immune response).
- Healthy European adults received two injections (6 months apart) of either one of the formulations of Vi-CRM$_{197}$+O:2-CRM$_{197}$ or a control vaccine - a licensed vaccine against *Salmonella* Typhi (control for the first injections) and a licensed vaccine against diphtheria, tetanus, and pertussis (control for the second injections).
- We assessed the safety and reactogenicity as well as immune responses generated by the different Vi-CRM$_{197}$+O:2-CRM$_{197}$ formulations and by the control vaccines.
- There were no serious adverse reactions and no safety concerns with any of the vaccine formulations tested in the study. All Vi-CRM$_{197}$+O:2-CRM$_{197}$ formulations showed a comparable safety profile, which was also similar to the control vaccines.
- After the first injection, all Vi-CRM$_{197}$+O:2-CRM$_{197}$ formulations generated good immune responses against both *Salmonella* Typhi and *Salmonella* Paratyphi A. The response against *Salmonella* Typhi was higher than the one elicited by the licensed vaccine against *Salmonella* Typhi. The second vaccine injection did not further increase these immune responses.

What is the impact?

- Our findings indicate that there were no safety concerns with any formulation of Vi-CRM$_{197}$+O:2-CRM$_{197}$. A single vaccine dose was immunogenic for all vaccine formulations.
- These results support further clinical testing of the selected Vi-CRM$_{197}$+O:2-CRM$_{197}$ vaccine formulation (full dose without adjuvant) to help prevent both typhoid and paratyphoid fever in all age groups.

Supplementary methods

Inclusion criteria

Study participants were healthy adults (aged 18 to 50 years) as established by medical history, clinical examination, and screening laboratory investigations, who were seronegative for HIV, hepatitis B, and hepatitis C at screening, and who, in the opinion of the investigator, could comply with the protocol requirements. Female participants of childbearing potential were eligible for the study if they had practiced adequate contraception for 1 month prior to study intervention administration, had a negative pregnancy test on the day of study intervention administration, and had agreed to continue adequate contraception during the entire treatment period and for 1 month after the last study intervention administration.

Exclusion criteria

Participants were not included in the study if they met any of the following criteria:

Medical conditions:
1. Progressive, unstable, or uncontrolled clinical conditions.
2. History of any reaction or hypersensitivity likely to be exacerbated by any component of the vaccine.
3. Hypersensitivity, including allergy, to medicinal products or medical equipment whose use was foreseen in this study.
4. Clinical conditions representing a contraindication to intramuscular vaccination and blood draws.
5. Any confirmed or suspected immunosuppressive or immunodeficient condition, based on medical history and physical examination (no laboratory testing required).
6. Any behavioral or cognitive impairment or psychiatric disease that, in the opinion of the investigator, might interfere with the participant's ability to participate in the study.
7. Acute or chronic illness, clinically significant pulmonary, cardiovascular, hepatic, or renal functional abnormality, as determined by physical examination or laboratory screening tests. Participants with a minor illness (such as mild diarrhea or mild upper respiratory infection) without fever might be enrolled at the discretion of the investigator.
8. Any clinically significant hematological (hemoglobin level, white blood cell, lymphocyte, neutrophil, eosinophil, platelet, red blood cell count, and erythrocyte mean corpuscular volume) and/or biochemical (alanine aminotransferase, aspartate aminotransferase, creatinine, and total protein) laboratory abnormality. The investigator used their clinical judgement to decide which abnormalities were clinically significant.
9. Confirmed positive COVID-19 test during the period starting 14 days before the first administration of study vaccines (Day [D] -14 to D1).
10. Any other clinical condition that, in the opinion of the investigator, might have posed additional risk to the participant due to participation in the study.
11. Confirmed or suspected autoimmune diseases (e.g., vitiligo, autoimmune thyroiditis).

Prior/concomitant therapy
12. Previous administration of any type of typhoid vaccine (a live-attenuated typhoid vaccine [Ty21a], a Vi polysaccharide vaccine [Vi-PS], or a typhoid conjugate vaccine).
13. Use of any investigational or non-registered product (drug, vaccine, or medical device) other than the study interventions during the period starting 30 days before the first administration of study vaccines (D -30 to D1), or planned use during the study period.
14. A vaccine not foreseen by the study protocol administered during the period starting at 14 days before the first dose (21 days in the case of live vaccines) and ending 28 days after the last dose of study intervention administration, with the exception of flu and COVID-19 vaccines, administered during the period starting at 7 days before and 7 days after each dose (14 days before and 14 days after in case of live vaccines). In case emergency mass vaccination for an unforeseen public health threat (e.g., a pandemic) was recommended and/or organized by public health authorities outside the routine authorization program, the time period described above could be reduced if necessary for that vaccine, provided it was used according to the local governmental recommendations and that the Sponsor was notified accordingly. When regulations allowed, the recommended time intervals for administration of those vaccines were at least 7 days before or 7 days after (at least 14 days before or 14 days after in case of live vaccines) each dose of study intervention administration.
15. Administration of long-acting immune-modifying drugs at any time during the study period (e.g., infliximab).
16. Administration of immunoglobulins and/or any blood products or plasma derivatives during the period starting 3 months before the administration of the first dose of study intervention or planned administration during the study period.

17. Chronic administration (defined as more than 14 days in total) of immunosuppressants or other immune-modifying drugs during the period starting 3 months prior to the first study intervention dose. For corticosteroids, this meant prednisone equivalent ≥20 mg/day for adult participants. Inhaled and topical steroids were allowed.

Prior/concurrent Clinical Study Experience

18. Concurrently participating in another clinical study, at any time during the study period, in which the participant had been or would be exposed to an investigational or a non-investigational vaccine/product (drug/invasive medical device).

Other exclusion criteria

19. History of travel to countries of Asia that were considered endemic for enteric fever in the last 3 years and during the study duration.
20. Pregnant or lactating female.
21. Female participants who were planning to become pregnant or planning to discontinue contraceptive precautions.
22. History of or current chronic alcohol consumption and/or drug abuse; chronic alcohol consumption was defined as one or more of the following: a) a prolonged period of frequent and heavy alcohol use, b) the inability to control drinking once it has begun, c) physical dependence manifested by withdrawal symptoms when the individual stops using alcohol, d) tolerance or the need to use increasing amounts of alcohol to achieve the same effects, and e) a variety of social and/or legal problems arising from alcohol use.
23. Any study personnel or immediate dependents, family, or household member.

Supplementary table S1. Study groups and intervention

	N	Study interventions
**Study groups**		
Step 1a: Low-dose without Alum	12	2 vaccinations (D1 and D169) with low dose of Vi-CRM_{197}+O:2-CRM_{197} without Alum
Step 1a: Control	6	1 vaccination (D1) with Vi-PS and 1 vaccination (D169) with DTaP
Step 1b: Low-dose with Alum	12	2 vaccinations (D1 and D169) with low dose of Vi-CRM_{197}+O:2-CRM_{197} with Alum
Step 1b: Control	6	1 vaccination (D1) with Vi-PS and 1 vaccination (D169) with DTaP
Step 2: Full-dose without Alum	24	2 vaccinations (D1 and D169) with full dose of Vi-CRM_{197}+O:2-CRM_{197} without Alum
Step 2: Full-dose with Alum	24	2 vaccinations (D1 and D169) with full dose of Vi-CRM_{197}+O:2-CRM_{197} with Alum
Step 2: Control	12	1 vaccination (D1) with Vi-PS and 1 vaccination (D169) with DTaP

Alum, aluminium hydroxide; CRM_{197}, Cross-Reacting Material 197; D, day; DTaP, diphtheria toxoid-tetanus toxoid-acellular pertussis (adsorbed) vaccine; N, number of participants; Vi-PS, typhoid Vi polysaccharide vaccine

Supplementary figure S1. Study design

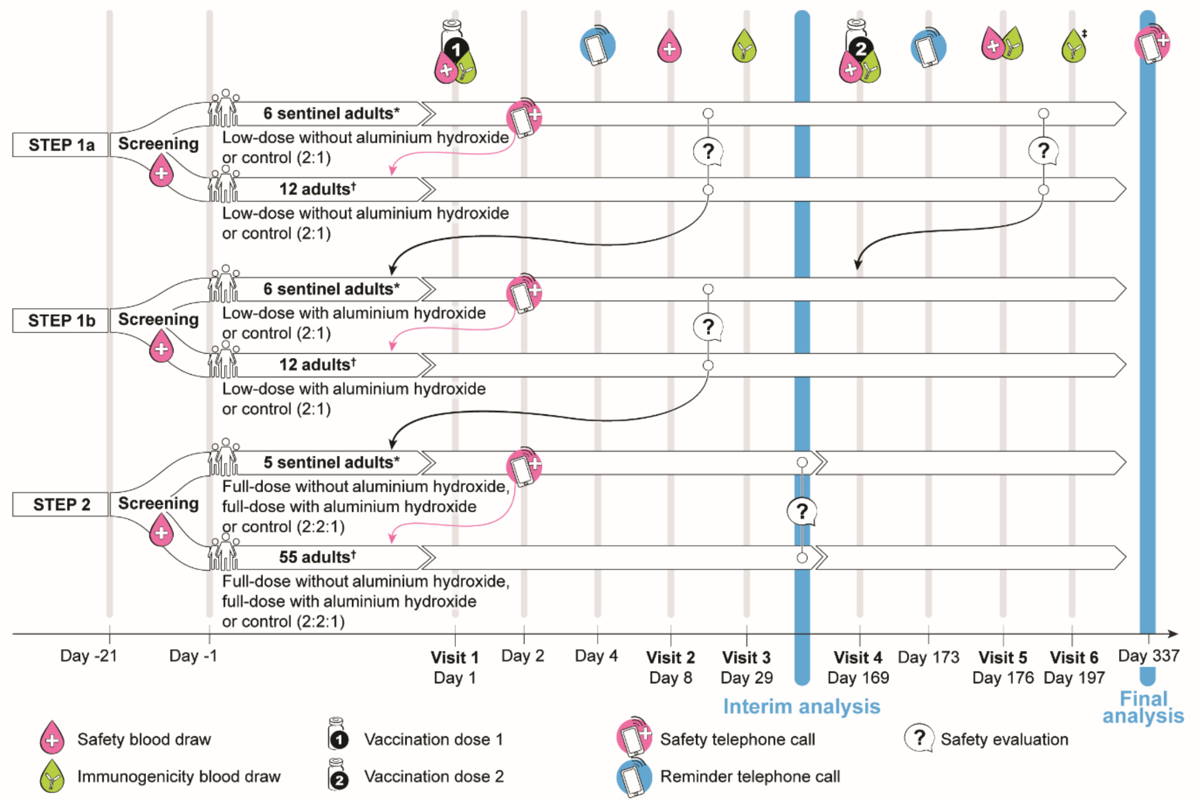

*Participants administered the first study dose consecutively on separate days.
†Participants administered study dose consecutively with intervals of at least 60 minutes.
‡An additional blood sample was collected for assay standard generation.

Typhoid Vi polysaccharide vaccine (Vi-PS; *Typhim Vi*, Sanofi Pasteur) was used as a control for the first vaccination on day 1, whereas tetanus toxoid, reduced diphtheria toxoid, and acellular pertussis vaccine, adsorbed (DTaP; *Boostrix*, GSK) was used as a control for the second vaccination on day 169.

Details of intervention administration

For the first study intervention administration, participants were vaccinated sequentially. At day (D) 1, sentinel participants received the first study intervention 1 day apart and non-sentinel participants received it at least 1 hour apart. At D169, all participants received the second study intervention at least 1 hour apart. All study participants were observed closely for at least 1 hour after the administration of the study vaccines. Both the investigational and the control vaccines were administered intramuscularly into the deltoid, upper, non-dominant arm.

Vaccine preparation and administration was handled by unblinded center staff at a different location from where clinical assessment and evaluation were performed.

Supplementary table S2. Safety screening laboratory assays and pre-/post-vaccination safety laboratory assays

Laboratory assessments	Parameters
**Hematology***	• WBC<br>• RBC<br>• Hemoglobin<br>• Hematocrit<br>• Platelets<br>• Eosinophils<br>• Basophils<br>• Neutrophils<br>• Monocytes<br>• Lymphocytes
**Clinical chemistry**	• Total bilirubin<br>• AST*<br>• ALT*<br>• γ-glutamyl transferase<br>• LDH<br>• AP<br>• Total proteins<br>• Glucose (random glucose)<br>• BU*#<br>• Creatinine*<br>• Sodium<br>• Potassium
**Other screening tests**	• Hepatitis B surface antigen antibodies<br>• Hepatitis C virus antibodies<br>• HIV antibodies<br>• Prothrombin time<br>• Blood hCG pregnancy test (for women of childbearing potential)
**Other pre-/post-vaccination safety laboratory assays**	• Urine hCG pregnancy test (as needed for women of childbearing potential) required at Visit 1 and Visit 4

ALT, alanine aminotransferase; AP, alkaline phosphatase; AST, aspartate aminotransferase; BU, blood urea; hCG, human chorionic gonadotropin; HIV, human immunodeficiency virus; LDH, lactate dehydrogenase; RBC, red blood cells; WBC, white blood cells

*The parameters were assessed at both screening and pre-/post-vaccination periods. Pre-/post-vaccination safety laboratory parameters were assessed at Visits 1 and 2 for the first dose administered and at Visit 4 and Visit 5 for the second dose administered.

#Blood urea was converted to blood urea nitrogen for grading purposes.

All hematology assay outputs were reported in absolute values. All events of ALT ≥3 × upper limit of normal (ULN) and bilirubin ≥2 × ULN (>35·0% direct bilirubin) or ALT ≥3 × ULN, which could indicate severe liver injury (possible Hy’s Law), were reported as serious adverse events.

Definitions of adverse events

An adverse event (AE) was defined as any untoward medical occurrence (an unfavorable/unintended sign including an abnormal laboratory finding), symptom, or disease (new or exacerbated) in a clinical study participant that was temporally associated with the study intervention, including:

- Significant or unexpected worsening or exacerbation of the condition/indication under study.
- Exacerbation of a chronic or intermittent pre-existing condition including either an increase in frequency and/or intensity of the condition.
- New conditions detected or diagnosed after administration of the study intervention even though they may have been present before study start.
- Signs, symptoms, or the clinical sequelae of a suspected drug, disease, or other interaction.
- Signs, symptoms, or the clinical sequelae of a suspected overdose of either the study intervention or a concurrent medication.
- Signs or symptoms temporally associated with administration of the study intervention.
- Signs, symptoms that required medical attention (e.g., hospital stays, physician visits, and emergency room visits).
- Significant failure of an expected pharmacologic or biological action.
- Pre- or post- intervention events that occurred as a result of protocol-mandated procedures (i.e., invasive procedures, modification of participant’s previous therapeutic regimen).
- Clinically significant abnormal laboratory findings or other abnormal assessments that were present at baseline and significantly worsened following the start of the study were also reported as AEs or serious AEs (SAEs).

An SAE was defined as any untoward medical occurrence that:

- Resulted in death.
- Was life-threatening.
- Required hospitalization or prolongation of existing hospitalization.
- Resulted in disability/incapacity.
- Was a congenital anomaly/birth defect in the offspring of a study participant.

a. Abnormal pregnancy outcomes (e.g., spontaneous abortion, fetal death, stillbirth, congenital anomalies, ectopic pregnancy).
b. Other situations: Medical or scientific judgment had to be exercised in deciding whether reporting was appropriate in other situations. Important medical events that were not immediately life-threatening or did not result in death or hospitalization but jeopardized the participant or required medical or surgical intervention to prevent one of the other outcomes listed in the above definition had to be considered serious. Examples of such events were invasive or malignant cancers; intensive treatment in an emergency room or at home for allergic bronchospasm; blood dyscrasias; and convulsions that did not result in hospitalization.

Solicited events were events recorded as endpoints in this study. The presence/occurrence/intensity of these events was actively solicited from the participant or an observer during a specified follow-up period following study intervention administration (i.e., during 7 days after each vaccination). The intensity of the solicited AEs was assessed as described in Supplementary table S3 below.

An unsolicited AE was defined as an AE that was either not included in the list of solicited events or could be included in the list of solicited events but with an onset outside the specified period of follow-up for solicited events. The maximum intensity that occurred over the duration of an event for all unsolicited AEs (including SAEs) recorded during the study was assessed by the investigator, according to one of three categories as described in Supplementary table S4 below.

Supplementary table S3. Intensity scales for solicited adverse events

Event	Intensity grade	Parameter
	Administration-site events	
Pain at administration site	0	None
	1	Mild: Any pain neither interfering with nor preventing normal everyday activities
	2	Moderate: Painful when limb is moved and interferes with everyday activities
	3	Severe: Significant pain at rest. Prevents normal everyday activities
Redness/swelling at administration site	0	Absent: greatest surface diameter <25 mm
	1	Mild: ≥25-≤50 mm
	2	Moderate: >50-≤100 mm
	3	Severe: >100 mm
	Systemic events	
Fever	0	Absent: <38·0°C
	1	Mild: ≥38·0°C-<38·5°C
	2	Moderate: ≥38·5°C-<39·0°C
	3	Severe: ≥39·0°C
Headache	0	None
	1	Mild: Headache that is easily tolerated
	2	Moderate: Headache that interferes with normal activity
	3	Severe: Headache that prevents normal activity
Fatigue (tiredness)	0	Normal
	1	Mild: Fatigue that is easily tolerated
	2	Moderate: Fatigue that interferes with normal activity
	3	Severe: Fatigue that prevents normal activity
Myalgia (muscle pain)	0	None
	1	Mild: Myalgia present but does not interfere with activity
	2	Moderate: Myalgia that interferes with normal activity
	3	Severe: Myalgia that prevents normal activity
Arthralgia (joint pain)	0	None
	1	Mild: Arthralgia present but does not interfere with activity
	2	Moderate: Arthralgia that interferes with normal activity
	3	Severe: Arthralgia that prevents normal activity

Supplementary table S4. Intensity categories for unsolicited adverse events

1 (mild)	An AE that is easily tolerated by the participant, causing minimal discomfort, and not interfering with everyday activities.
2 (moderate)	An AE that is sufficiently discomforting to interfere with normal everyday activities.
3 (severe)	An AE that prevents normal, everyday activities (such an AE would, for example, prevent attendance at work and would necessitate the administration of corrective therapy).

AE, adverse event

Supplementary table S5. Toxicity grading scale for safety laboratory assessments

Laboratory test	Grade 1	Grade 2	Grade 3	Grade 4
BUN, mg/dL	<23-26	>26-31	>31	Requires dialysis
Creatinine, mg/dL (female)	>1·1-1·7	>1·7-2·0	>2·0-2·5	>2·5 or requires dialysis
Creatinine, mg/dL (male)	>1·3-1·7	>1·7-2·0	>2·0-2·5	>2·5 or requires dialysis
Eosinophils, cell/mm^3	>500-1500	>1500-5000	>5000	Hypereosinophilic
Hemoglobin (female), g/dL	11·0-<11·6	9·5-<11·0	8·0-<9·5	<8·0
Hemoglobin (female/male) - change from baseline value, g/dL	Any decrease-1·5	>1·5-2·0	>2·0-5·0	>5·0
Hemoglobin (male), g/dL	12·5-<12·9	10·5-<12·5	8·5-<10·5	<8·5
Liver function tests - ALT, AST increase by factor	≥1·1-2·5 x ULN	>2·5-5·0 x ULN	>5·0-10 x ULN	>10 x ULN
Lymphocyte decrease, cell/mm^3	750-<800	500-<750	250-<500	<250
Neutrophil decrease, cell/mm^3	1340	1000-<1340	500-<1000	<500
Platelet decrease, cell/mm^3	125 000-<150 000	100 000-<125 000	25 000-<100 000	<25 000
WBC decrease, cell/mm^3	2500-<3700	1500-<2500	1000-<1500	<1000
WBC increase, cell/mm^3	>10 000-15 000	>15 000-20 000	>20 000-25 000	>25 000

ALT, alanine aminotransferase; AST, aspartate aminotransferase; BUN, blood urea nitrogen; ULN, upper limit of normal; WBC, white blood cells

Toxicity grading scale for safety laboratory test results was based on the US FDA Guidance for Industry[1] and adjusted in consideration of local normal ranges provided by the site. Parameters not included in the FDA grading scales were not graded; their assessment was based on laboratory normal ranges and medical judgment.

Details of immunological assays

Anti-Vi specific immunoglobulin (Ig) G antibodies (in µg/mL equivalents) were measured by a qualified, fit-for-purpose enzyme-linked immunosorbent assay (ELISA), with a lower limit of quantification (LLoQ) of 2·2 µg/mL.[2] Anti-O:2 IgG antibodies were measured by an ELISA which used a standard curve with an assigned value of ELISA units (EU)/mL, using O:2 as coating antigen, with a lower limit of quantification of 13·3 EU/mL.[3] Immune responses to the paratyphoid A component of the vaccine formulations were assessed using a luminescence serum bactericidal assay (SBA) and expressed in serum titers, defined as serum dilutions giving 50% inhibition of bacterial growth (IC_{50}). The SBA lower limit of quantification was 27·3 IC_{50}.[3]

Supplementary table S6. Study objectives and endpoints

Objectives	Endpoints
**Primary**	
Evaluate the safety profile of the Vi-CRM$_{197}$+O:2-CRM$_{197}$ vaccine, with and without adjuvant.	• Percentage of participants with solicited administration-site AEs during 7 days after each dose, on the days of vaccination and the 6 subsequent days (study intervention administered on D1 and D169), per study group.<br>• Percentage of participants with solicited systemic AEs during 7 days after each dose, on the days of vaccination and the 6 subsequent days (study intervention administered on D1 and D169), per study group.<br>• Percentage of participants with unsolicited AEs during 28 days after each dose, on the days of vaccination and 27 subsequent days (study intervention administered on D1 and D169), per study group.<br>• Percentage of participants with any SAE from first dose until 28 days after second dose (D1 to D197), per study group.<br>• Percentage of participants with AEs/SAEs leading to withdrawal from the study or withholding further study intervention administration, from first dose administration until 28 days after second dose administration (D1 to D197), per study group.<br>• Percentage of participants with deviations from normal or baseline[†] values of hematological, renal, and hepatic panel test results at 7 days after each dose (D8 and D176), per study group.
**Secondary**	
Evaluate the long-term safety profile of the Vi-CRM$_{197}$+O:2-CRM$_{197}$ vaccine, with and without adjuvant.	• Percentage of participants with any SAE from 28 days after the second dose (D197) up to D337, per study group.<br>• Percentage of participants with AEs/SAEs leading to withdrawal from the study from 28 days after the second dose (D197) up to D337, per study group.
Evaluate the immunogenicity profile of the typhoid and paratyphoid A components of the Vi-CRM$_{197}$+O:2-CRM$_{197}$ vaccine, with and without adjuvant, using ELISA.	• GMC of anti-Vi antigen IgG antibody concentrations as measured by ELISA, before first dose (D1), 28 days after first dose (D29), before second dose (D169), 7 days after second dose (D176), and 28 days after second dose (D197), per study group.<br>• GMC of anti-O:2 IgG antibody concentrations, as measured by ELISA, before first dose (D1), 28 days after first dose (D29), before second dose (D169), 7 days after second dose (D176), and 28 days after second dose (D197), per study group.
Evaluate different seroresponse rates to the typhoid component and the paratyphoid A component of the Vi-CRM$_{197}$+O:2-CRM$_{197}$ vaccine, with and without adjuvant.	• Percentage of participants achieving anti-Vi antigen IgG antibody concentrations ≥4·3 µg/mL*, as measured by ELISA, before first dose (D1), 28 days after first dose (D29), before second dose (D169), 7 days after second dose (D176), and 28 days after second dose (D197), per study group.<br>• Percentage of participants achieving anti-Vi antigen IgG antibody concentrations ≥2·0 µg/mL**, as measured by ELISA, before first dose (D1), 28 days after first dose (D29), before second dose (D169), 7 days after second dose (D176), and 28 days after second dose (D197), per study group.<br>• Percentage of participants achieving at least 4-fold*** increase in anti-O:2 IgG antibody concentrations, as measured by ELISA, at 28 days after first dose (D29), before second dose (D169), 7 days after second dose (D176), and 28 days after second dose (D197) compared to first vaccination baseline (D1), per study group.<br><br>*This threshold, estimated to be protective against typhoid fever, was established in previous studies[4] and then used in the registration of Vi conjugated vaccine in India.[5]<br>**This threshold, estimated to be protective against typhoid fever, was defined as a short-term threshold in a previous study.[6]<br>***This threshold was chosen as a clinically meaningful threshold that, in the absence of an established correlate of protection, could allow quantitative decisions to be made for further development of this component of the vaccine.
**Tertiary**	
Evaluate the immunogenicity profile of the paratyphoid A component of the Vi-CRM$_{197}$+O:2-CRM$_{197}$ vaccine, with and without adjuvant, using SBA.	• GMT of serum bactericidal activity against a selected panel of *Salmonella* Paratyphi A strains, as measured by SBA, before first dose (D1), 28 days after first dose (D29), before second dose (D169), 7 days after second dose (D176), and 28 days after second dose (D197), per study group.
To further explore immune responses to the typhoid	• Antibody features (subclassing, affinity, avidity) and functional

Objectives	Endpoints
and paratyphoid components of the Vi-CRM197+O:2-CRM_{197} vaccine with and without adjuvant in a subset of participants.[§]	properties (induction of cellular phagocytosis and cellular cytotoxicity, natural killer-cell activation, eosinophil and basophil degranulation, complement deposition, neutrophil activation/phagocytosis, dendritic cell phagocytosis, mucin binding, glycosylation, and Fc receptor alteration), as measured by systems serology analysis.

AE, adverse event; CRM_{197}, Cross-Reacting Material 197; D, day; ELISA, enzyme-linked immunosorbent assay; GMC, geometric mean concentration; GMT, geometric mean titer; IgG, immunoglobulin G; SAE, serious adverse event; SBA, serum bactericidal assay

[†]Baseline for post-first vaccination at D8 was D1 (could also be screening blood draw, if performed within 3 days of first vaccination), and baseline for post-second vaccination at D176 was D169.

[§]At the time of this manuscript, results of respective tertiary objective and the endpoints were not yet available.

Details of statistical analyses

The sample size for the safety analyses was based on the assumption that in study Steps 1a and 1b, a sample of 12 participants receiving the investigational vaccine would provide a probability of at least 80% to observe at least one AE, if the true AE rate was ≥15·0%; and in Step 2, a sample of 24 participants receiving the investigational vaccine would provide a probability of at least 80% to observe at least one AE, if the true AE rate was ≥7·5%, estimated with the PROBBNML function from SAS 9.4. The table below shows the probability to observe at least one AE for different sample sizes and AE rates.

N receiving investigational vaccine	True AE rate (%)	Probability to observe at least one AE (%)
12	1·0	11·4
	2·5	26·2
	5·0	46·0
	7·5	60·8
	10·0	71·8
	12·5	79·9
	15·0	85·8
	17·5	90·1
	20·0	93·1
24	1·0	21·4
	2·5	45·5
	5·0	70·8
	7·5	84·6
	10·0	92·0
	12·5	95·9
	15·0	98·0
	17·5	99·0
	20·0	99·5

AE, adverse event; N, number of participants

The sample size for the immunogenicity analyses was based on the percentage of participants with 4-fold increases in anti-O:2 IgG antibody concentrations, defined as a measure of a seroresponse. The paratyphoid component was chosen for the expected seroresponse as, in contrast to typhoid vaccines, currently, no approved vaccines against paratyphoid fever are available. Results for both Full-dose groups (adjuvanted and unadjuvanted) were used to make decisions for the planning of subsequent studies and the clinical development program. Assuming a 12% participant dropout rate among 24 participants from each Full-dose group, 21 participants per group were considered evaluable. The table below shows precision in terms of 95% confidence interval (CI) on the 4-fold increase in these participants, estimated using PASS 2019, v 19.0.1 Two-Sided Confidence Intervals for One Proportion with Exact Formula (Clopper-Pearson).).

N receiving investigational vaccine	N (%) with 4-fold increases in anti-O:2 IgG	95% confidence interval
21	10 (47·6)	25·7-70·2
	11 (52·4)	29·8-74·3
	12 (57·1)	34·0-78·2
	13 (61·9)	38·4-81·9
	14 (66·7)	43·0-85·4
	15 (71·4)	47·8-88·7
	16 (76·2)	52·8-91·8
	17 (81·0)	58·1-94·6
	18 (85·7)	63·7-97·0

IgG, immunoglobulin G; N (%), number (percentage) of participants

The Exposed Set included all eligible participants from the Enrolled Set, i.e., all participants who entered the study (were randomized, or received study intervention, or underwent a post-screening study procedure) and who received at least one dose of the study intervention.

The Solicited Safety Set included all participants who received at least one dose of the study intervention and who had available solicited safety data.

The Unsolicited Safety Set included all participants who received at least one dose of the study intervention and reported having/not having unsolicited adverse events (AEs) following their enrollment in the study up to 28 days after vaccination.

The Full Analysis Set (FAS) included all participants who received at least one dose of the study intervention and had available post-vaccination immunogenicity data. The allocation in a group was based on the randomized intervention. The FAS for immunogenicity was defined by timepoint.

The Per-Protocol Set (PPS) included all eligible participants who received each dose as per-protocol, had available immunogenicity results post-dose, complied with dosing/blood draw intervals, without intercurrent conditions that might interfere with immunogenicity, without prohibited concomitant medication/vaccination, and without protocol deviation leading to exclusion. The PPS for immunogenicity was defined by timepoint.

Baseline was defined as:

- Day (D) 1 pre-dose for ≥4-fold increase calculation.
- D1 and each vaccination pre-dose for immunogenicity within-participant geometric mean ratio/ geometric mean fold-rise.
- Each vaccination pre-dose for hematology and biochemistry safety assessments.

For immunogenicity measurements, fold-change from baseline was calculated as post-vaccination value/baseline value.

The geometric mean concentration/titer calculations were performed by taking the anti-log of the mean of the $\log_{10}$ concentration/titer transformations. The CI for geometric means was derived by raising 10 to the CI associated with the mean of $\log_{10}$ values, i.e., CI of geometric mean = 10^(CI for the mean of $\log_{10}$ values). Participants whose antibody concentrations/titers were below the cut-offs of the assays were given an arbitrary value of half the cut-offs for the purpose of the analyses.

The above calculations were performed by LLOQ status at baseline (above or equal to cut-off versus below the cut-off) for both anti-Vi and anti-O:2 antibodies.

The following derivation rules were applied in the context of LLOQ values:

If baseline (D1) value was ≥LLOQ:

- 4-fold was defined as 4 times the baseline value
- within-participant geometric mean ratio (GMR) was defined as within-participant GMR against the baseline value.

If baseline (D1) value was <LLOQ:

- 4-fold was defined as 4 times the LLOQ cut-off value
- GMR was defined as within-participant GMR against LLOQ/2.

Antibody concentrations/titers were visually represented using reverse cumulative distribution curves in the PPS for the *Salmonella* Typhi (measured by ELISA), and for the *Salmonella* Paratyphi A components (measured by both ELISA and serum bactericidal assay).

Quantitative variables were summarized using descriptive statistics, including the number (sample size or available data), mean, and standard deviation.

The exact 2-sided 95% CIs for a proportion within an intervention group were calculated using the Clopper-Pearson exact method.

Supplementary results

Supplementary figure S2. Detailed trial profile

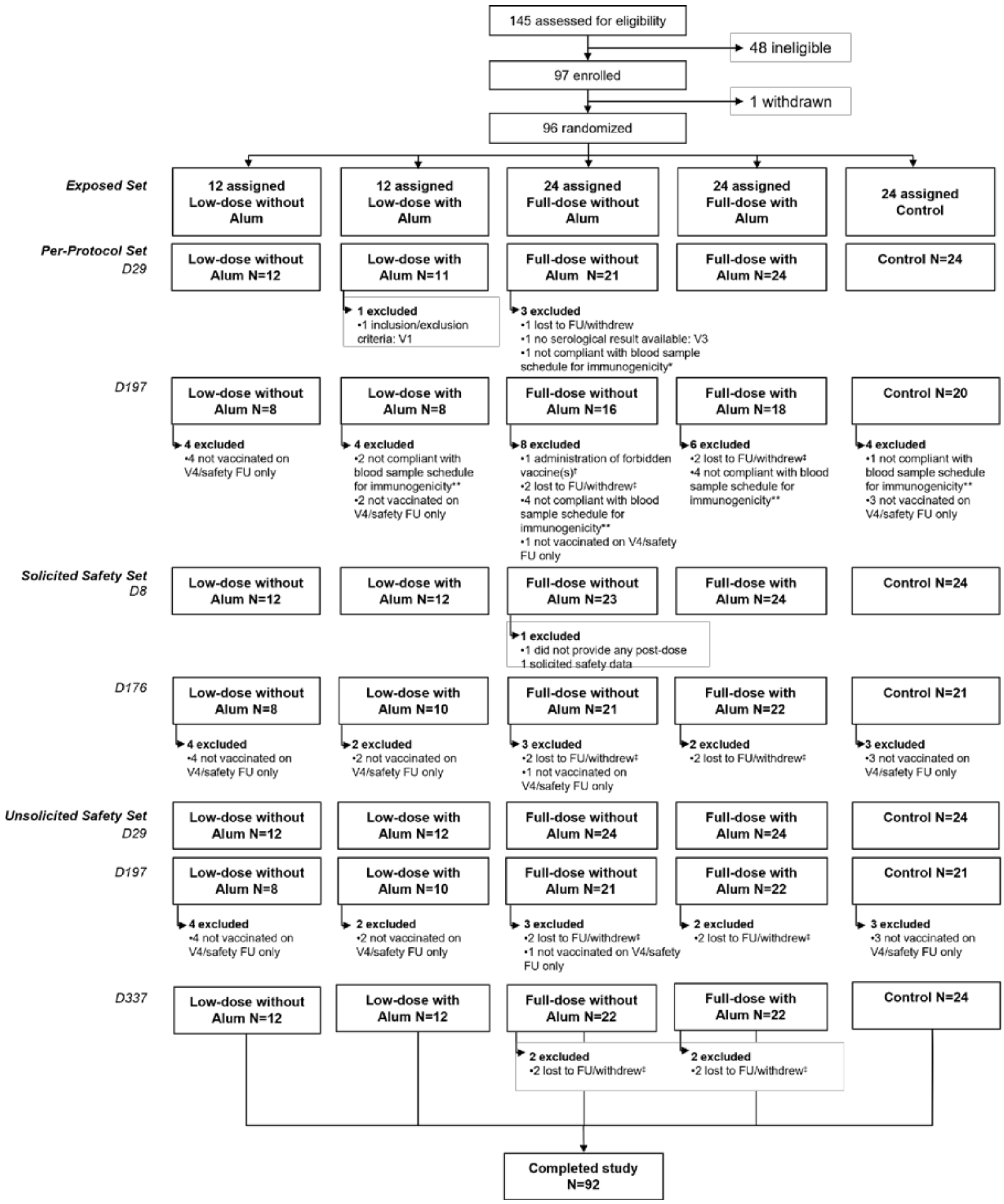

Alum, aluminium hydroxide; D, day; FU, follow-up; N, number of participants; V, visit
*Out of interval between V1 and V3.
**Out of interval between V4 and V6.
[†]Administration of concomitant vaccine(s) forbidden in the protocol: between V5 and V6.
[‡]1: withdrawal by the participant not due to adverse event; 1: lost to follow-up.

The number of participants in each group included in each analysis set is shown in boxes. Reasons for exclusion (and number of participants with the specific reason) are shown below the boxes, where applicable.

Supplementary table S7. Demographics and baseline characteristics of study participants in the Per-Protocol Set

	Low-dose without Alum (N=12)	Low-dose with Alum (N=11)	Full-dose without Alum (N=21)	Full-dose with Alum (N=24)	Control (N=24)	Total (N=92)
**Post-dose 1 (D29)**						
**Age (years), mean (SD)**	31·9 (10·8)	31·9 (8·8)	27·1 (9·8)	27·1 (9·8)	30·7 (10·6)	29·2 (10·1)
**Sex, n (%)**						
Female	11 (91·7)	5 (45·5)	14 (66·7)	17 (70·8)	18 (75·0)	65 (70·7)
Male	1 (8·3)	6 (54·5)	7 (33·3)	7 (29·2)	6 (25·0)	27 (29·3)
**Height (cm), mean (SD)**	168·6 (3·9)	174·4 (11·2)	172·2 (10·2)	173·7 (7·7)	169·5 (6·5)	171·7 (8·3)
**Weight (kg), mean (SD)**	68·7 (10·3)	80·1 (13·4)	73·0 (10·8)	71·3 (13·2)	71·4 (13·3)	72·4 (12·5)
**BMI (kg/m^2), mean (SD)**	24·2 (3·6)	26·5 (4·8)	24·6 (3·6)	23·5 (3·4)	24·8 (4·5)	24·6 (4·0)
**Post-dose 2 (D197)**						
	**Low-dose without Alum (N=8)**	**Low-dose with Alum (N=8)**	**Full-dose without Alum (N=16)**	**Full-dose with Alum (N=18)**	**Control (N=20)**	**Total (N=70)**
**Age (years), mean (SD)**	34·5 (11·3)	33·0 (9·4)	28·1 (10·5)	27·7 (10·0)	31·0 (11·2)	30·1 (10·5)
**Sex, n (%)**						
Female	8 (100)	3 (37·5)	11 (68·8)	13 (72·2)	17 (85·0)	52 (74·3)
Male	0 (0·0)	5 (62·5)	5 (31·3)	5 (27·8)	3 (15·0)	18 (25·7)
**Height (cm), mean (SD)**	167·8 (4·3)	174·3 (9·7)	172·7 (9·7)	173·9 (8·2)	169·7 (6·9)	171·7 (8·2)
**Weight (kg), mean (SD)**	70·7 (11·4)	83·0 (12·2)	73·0 (12·0)	71·4 (12·5)	72·3 (14·1)	73·3 (12·9)
**BMI (kg/m^2), mean (SD)**	25·1 (3·8)	27·5 (4·5)	24·5 (3·9)	23·5 (3·2)	25·1 (4·9)	24·8 (4·1)

Alum, aluminium hydroxide; BMI, body mass index; D, day; N, number of participants in the group; n (%), number (percentage) of participants in a given category; SD, standard deviation
For all variables, no missing values were reported.
The Per-Protocol Set was defined by timepoint.

Summary of protocol deviations leading to elimination from any analyses

In the Enrolled Set (N=97), six participants (6·2%) had critical protocol deviations: 4/12 (33·3%) in the Low-dose without Alum, 1/12 (8·3%) in Low-dose with Alum, and 1/24 (4·2%) in the Control group. All the critical protocol deviations were related to inability to meet the exclusion criteria.
Twenty-three participants (23·7%) had major protocol deviations: 2/12 (16·7%) in the Low-dose with Alum, 7/24 (29·2%) in the Full-dose with Alum, 9/24 (37·5%) in the Full-dose without Alum, and 5/24 (20·8%) participants in the Control group. The reasons for major protocol deviations were: participants had administration of concomitant vaccine(s) that was (were) forbidden in the protocol, no serological results available, participants did not comply with blood sample schedule for immunogenicity evaluation, and participants did not comply with vaccination schedule.
In the Exposed Set (N=96), one participant (1·0%) had critical protocol deviations leading to exclusion from the PPS at D29 (1/12 [8·3%] in the Low-dose with Alum group) and five participants (5·2%) at D169 (4/12 [33·3%] in the Low-dose without Alum and 1/24 [4·2%] in the Control group).
There were no critical protocol deviations leading to exclusion from the PPS at the other timepoints.

Supplementary table S8. Summary of protocol deviations leading to exclusion from the PPS (Exposed Set; N=96)

	**Low-dose without Alum N=12** n (%)	**Low-dose with Alum N=12** n (%)	**Full-dose without Alum N=24** n (%)	**Full-dose with Alum N=24** n (%)	**Control N=24** n (%)
**PPS D29**					
No serological result available: V3	0 (0·0)	0 (0·0)	1 (4·2)	0 (0·0)	0 (0·0)
Non-compliance with blood sample schedule for immunogenicity evaluation*	0 (0·0)	0 (0·0)	1 (4·2)	0 (0·0)	0 (0·0)
**PPS D169**					
Laboratory assessment/no serological result available: V4	0 (0·0)	0 (0·0)	0 (0·0)	0 (0·0)	1 (4·2)
Non-compliance with the vaccination schedule**	0 (0·0)	0 (0·0)	0 (0·0)	0 (0·0)	1 (4·2)
**PPS D176**					
No serological result available: V5	0 (0·0)	0 (0·0)	1 (4·2)	1 (4·2)	0 (0·0)
Non-compliance with blood sample schedule for immunogenicity evaluation***	0 (0·0)	0 (0·0)	2 (8·3)	3 (12·5)	2 (8·3)
**PPS D197**					
Administration of concomitant vaccine(s) forbidden in the protocol between V5 and V6	0 (0·0)	0 (0·0)	1 (4·2)	0 (0·0)	0 (0·0)
Non-compliance with blood sample schedule for immunogenicity evaluation****	0 (0·0)	2 (16·7)	4 (16·7)	4 (16·7)	1 (4·2)

Alum, aluminium hydroxide; D, day; N, number of participants; n (%), number (percentage) of participants reporting the deviation at least once; PPS, Per-Protocol Set; V, visit

A participant experiencing multiple deviations was counted, at most, once per category and coded term.

*Out of interval between V1 and V3.

**Out of interval between V1 and V4.

***Out of interval between V4 and V5.

****Out of interval between V4 and V6.

Supplementary safety results

Supplementary figure S3. Summary of solicited events (Solicited Safety Set)

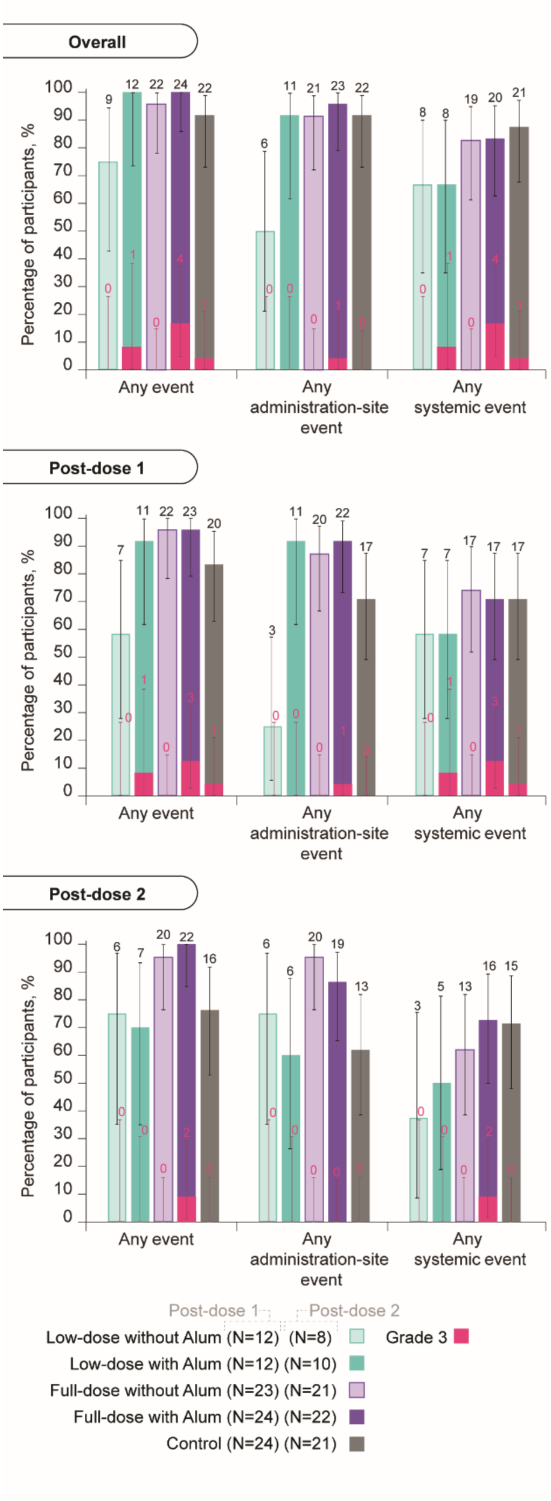

Alum, aluminium hydroxide; N, number of participants
The error bars represent 95% confidence intervals.
The numbers indicated above the error bars represent: in black - numbers of participants with events (any grade) per group, in pink - numbers of participants with grade 3 events per group.

Details of solicited events

Post-dose 1, pain was most common in the Low- and Full-dose with Alum groups, reported by 11/12 (92%; 95% CI 61·5-99·8) and 22/24 participants (92%; 73·0-99·0), respectively, followed by 20/23 (87%; 66·4-97·2) in the Full-dose without Alum, 17/24 (71%; 48·9-87·4) in the Control, and 3/12 (25%; 5·5-57·2) in the Low-dose without Alum group. Post-dose 2, pain was most common in the Full-dose groups (without Alum: 20/21 [95%; 76·2-99·9], with Alum: 19/22 participants [86%; 65·1-97·1]), followed by Low-dose without Alum (6/8 [75%; 34·9-96·8]), Control (13/21 [62%; 38·4-81·9]), and Low-dose with Alum group (6/10 [60%; 26·2-87·8]).

Post-dose 1, the incidence of fatigue ranged from 3/12 (25%; 95% CI 5·5-57·2) in the Low-dose with Alum to 12/24 participants (50%; 29·1-70·9) in the Full-dose with Alum group, and post-dose 2, from 1/8 (13%; 0·3-52·7) in the Low-dose without Alum to 9/21 participants (43%; 21·8-66·0) in the Control group. Post-dose 1, the incidence of headache ranged from 6/24 (25%; 9·8-46·7) in the Full-dose with Alum to 11/24 participants (46%; 25·6-67·2) in the Control group, and post-dose 2, from 2/8 (25%; 3·2-65·1) in the Low-dose without Alum to 9/21 participants (43%; 21·8-66·0) in both Full-dose without Alum and the Control group. Post-dose 1, the incidence of myalgia ranged from 3/12 (25%; 5·5-57·2) in both Low-dose groups to 15/24 participants (63%; 40·6-81·2) in the Full-dose with Alum group, and post-dose 2, from 3/10 (30%; 6·7-65·2) in the Low-dose with Alum to 9/21 participants (43%; 21·8-66·0) in both the Full-dose without Alum and the Control group.

Supplementary figure S4. Summary of unsolicited adverse events (Unsolicited Safety Set)

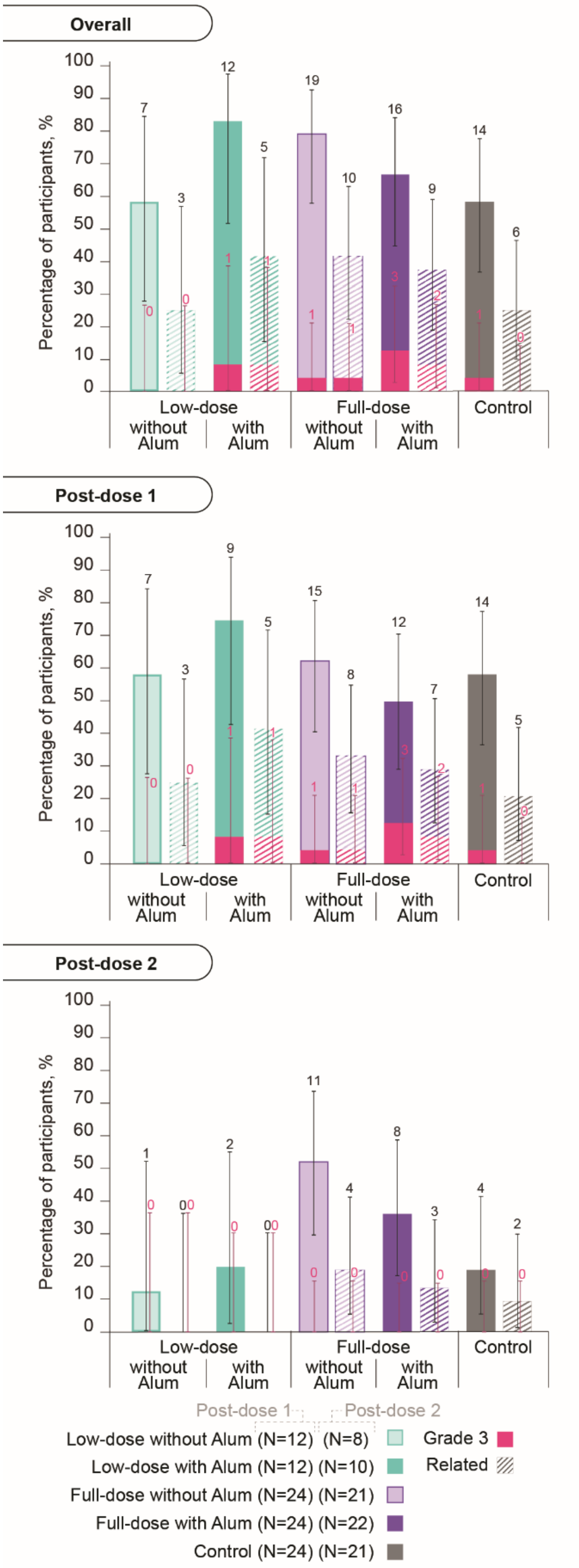

Alum, aluminium hydroxide; N, number of participants
The error bars represent 95% confidence intervals.
The numbers indicated above the error bars represent: in black - numbers of participants with AEs (any grade) per group, in pink - numbers of participants with grade 3 AEs per group.

Details of unsolicited AEs

Unsolicited adverse events considered related to vaccination occurring in at least two participants across the groups included: musculoskeletal stiffness (four participants: two [8·3%] in each Full-dose group), diarrhea (four participants: two [8·3%] in Full-dose without Alum, one [8·3%] in Low-dose with Alum, and one [4·2%] in Full-dose with Alum group), decreased hemoglobin level (three participants: one [4·2%] in Full-dose without Alum, and two [8·3%] in Full-dose with Alum group), nausea (three participants: one [4·2%-8·3%] in each Low-dose without Alum, Full-dose with Alum, and Control group), lymphadenopathy (two participants: one [4·2%] in each Full-dose without Alum and Control group), injection-site hemorrhage (two participants: one [8·3%] in Low-dose with Alum, and one [4·2%] in Full-dose without Alum group), and increased aspartate aminotransferase levels (two participants: one [4·2%] in each Full-dose without Alum and Control group).

Grade 3 unsolicited adverse events were: migraine (Low-dose with Alum: one participant [8·3%]), decreased hemoglobin level (Full-dose with Alum: two participants [8·3%]; Full-dose without Alum: one participant [4·2%]), cystitis (Full-dose with Alum: one participant [4·2%]), pharyngotonsillitis (Full-dose with Alum: one participant [4·2%]), and tooth abscess (Control: one participant [4·2%]), reported post-dose 1.

Two adverse events not related to vaccination, for which further study intervention administrations were withheld were: one case of lower abdominal pain (Low-dose with Alum; second dose withheld due to investigator’s decision) and one case of acute sinusitis (Control group; second dose withheld due to corticosteroid administration).

Supplementary immunogenicity results

Supplementary table S9. Anti-Vi IgG antibody GMCs, GMRs, and seroresponse rates as measured by ELISA (Per-Protocol Set)

	**Low-dose without Alum**			**Low-dose with Alum**			**Full-dose without Alum**			**Full-dose with Alum**			**Control**		
	**N**		**Value (95% CI)**	**N**		**Value (95% CI)**	**N**		**Value (95% CI)**	**N**		**Value (95% CI)**	**N**		**Value (95% CI)**
**GMC (µg/mL)**															
D1	12		1·10 (-)	11		1·52 (0·93-2·48)	21		1·14 (1·06-1·23)	24		1·17 (1·03-1·33)	24		1·10 (-)
D29	12		14·12 (8·21-24·29)	11		19·61 (9·10-42·28)	21		60·52 (35·28-103·82)	24		36·97 (20·56-66·48)	24		4·95 (3·23-7·59)
D169	8		4·36 (2·01-9·47)	10		11·17 (4·79-26·03)	21		18·58 (9·97-34·60)	22		12·55 (7·06-22·32)	21		6·18 (3·50-10·92)
D176	8		10·90 (4·72-25·21)	10		18·78 (9·05-38·97)	18		32·38 (19·82-52·89)	18		56·78 (38·23-84·32)	19		5·15 (3·02-8·77)
D197	8		10·16 (5·08-20·30)	8		18·88 (8·23-43·29)	16		33·75 (17·78-64·07)	18		37·14 (23·79-58·00)	20		5·17 (3·14-8·52)
**GMR**															
D29	12		12·84 (7·46-22·08)	11		12·90 (6·77-24·58)	21		53·01 (31·94-87·99)	24		31·55 (18·74-53·11)	24		4·50 (2·93-6·90)
D169	8		3·96 (1·82-8·61)	10		7·11 (3·51-14·40)	21		16·27 (8·94-29·61)	22		10·65 (6·59-17·22)	21		5·62 (3·18-9·93)
D176	8		9·91 (4·29-22·92)	10		11·96 (6·05-23·65)	18		28·19 (17·72-44·84)	18		47·45 (36·38-61·89)	19		4·68 (2·75-7·97)
D197	8		9·23 (4·62-18·46)	8		11·00 (5·15-23·49)	16		29·22 (15·71-54·35)	18		31·04 (23·14-41·65)	20		4·70 (2·85-7·74)
**Seroresponse*: participants with anti-Vi IgG concentration ≥4·3 µg/mL**															
	**N**	**n**	**% (95% CI)**	**N**	**n**	**% (95% CI)**	**N**	**n**	**% (95% CI)**	**N**	**n**	**% (95% CI)**	**N**	**n**	**% (95% CI)**
D1	12	0	0·0 (0·0-26·5)	9	0	0·0 (0·0-33·6)	21	0	0·0 (0·0-16·1)	23	0	0·0 (0·0-14·8)	24	0	0·0 (0·0-14·2)
D29	12	12	100 (73·5-100)	9	8	88·9 (51·8-99·7)	21	21	100 (83·9-100)	23	22	95·7 (78·1-99·9)	24	13	54·2 (32·8-74·4)
D169	8	6	75·0 (34·9-96·8)	8	7	87·5 (47·3-99·7)	21	20	95·2 (76·2-99·9)	21	17	81·0 (58·1-94·6)	21	13	61·9 (38·4-81·9)
D176	8	6	75·0 (34·9-96·8)	8	7	87·5 (47·3-99·7)	18	17	94·4 (72·7-99·9)	17	17	100 (80·5-100)	19	11	57·9 (33·5-79·7)
D197	8	7	87·5 (47·3-99·7)	6	5	83·3 (35·9-99·6)	16	15	93·8 (69·8-99·8)	17	17	100 (80·5-100)	20	12	60·0 (36·1-80·9)
**Seroresponse*: participants with anti-Vi IgG concentration ≥2·0 µg/mL**															
D1	12	0	0·0 (0·0-26·5)	9	0	0·0 (0·0-33·6)	20	0	0·0 (0·0-16·8)	23	0	0·0 (0·0-14·8)	24	0	0·0 (0·0-14·2)
D29	12	12	100 (73·5-100)	9	9	100 (66·4-100)	20	20	100 (83·2-100)	23	22	95·7 (78·1-99·9)	24	20	83·3 (62·6-95·3)
D169	8	6	75·0 (34·9-96·8)	8	7	87·5 (47·3-99·7)	20	19	95·0 (75·1-99·9)	21	21	100 (83·9-100)	21	17	81·0 (58·1-94·6)
D176	8	8	100 (63·1-100)	8	8	100 (63·1-100)	17	16	94·1 (71·3-99·9)	17	17	100 (80·5-100)	19	15	78·9 (54·4-93·9)
D197	8	8	100 (63·1-100)	6	6	100 (54·1-100)	15	15	100 (78·2-100)	17	17	100 (80·5-100)	20	16	80·0 (56·3-94·3)

Alum, aluminium hydroxide; CI, confidence interval; D, day; ELISA enzyme-linked immunosorbent assay; GMC, geometric mean concentration; GMR, within-participant geometric mean ratio comparing geometric mean to D1 pre-dose; IgG, immunoglobulin G; N, number of participants with available results in each category; n, number of participants with anti-Vi IgG concentration ≥2·0 µg/mL/≥4·3 µg/mL

*In participants seronegative (anti-Vi IgG antibody concentrations below 4·3 µg/mL or 2·0 µg/mL, respectively for each threshold) at baseline.

The Per-Protocol Set was defined by timepoint.

Supplementary figure S5. Reverse cumulative distribution curves of anti-Vi IgG by timepoint (Per-Protocol Set)

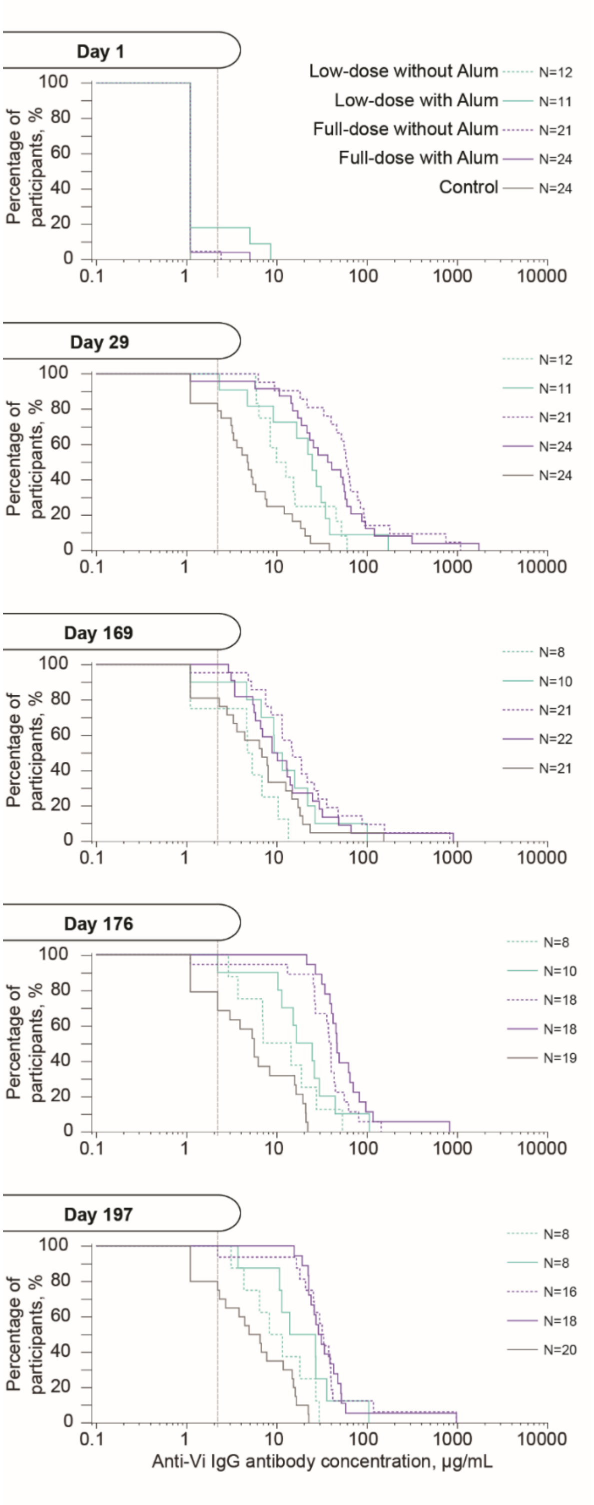

Alum, aluminium hydroxide; IgG, immunoglobulin G; N, number of participants
The dashed line represents the lower limit of quantification for anti-Vi IgG (2·2 μg/mL).
The Per-Protocol Set was defined by timepoint.

Supplementary table S10. Statistical comparison of immunogenicity variables – unadjusted and adjusted ratios for anti-Vi and anti-O:2 IgG antibodies, and bactericidal antibodies against *Salmonella* Paratyphi A (Per-Protocol Set)

Anti-Vi

Timepoint	Pairwise comparison	Unadjusted ratios			Adjusted ratios		
		Unadjusted GMR	95% CI	p-value	Adjusted GMR	95% CI	p-value
D29	Full-dose with Alum vs Full-dose without Alum	0·61	0·31-1·22	0·16	0·56	0·29-1·07	0·079
	Low-dose with Alum vs Low-dose without Alum	1·39	0·53-3·64	0·50	0·86	0·34-2·18	0·73
D169	Full-dose with Alum vs Full-dose without Alum	0·68	0·31-1·45	0·31	0·57	0·30-1·10	0·094
	Low-dose with Alum vs Low-dose without Alum	2·56	0·78-8·44	0·12	1·73	0·67-4·48	0·24
D176	Full-dose with Alum vs Full-dose without Alum	1·75	0·91-3·37	0·091	1·29	0·65-2·55	0·46
	Low-dose with Alum vs Low-dose without Alum	1·72	0·68-4·36	0·25	1·16	0·45-3·01	0·74
D197	Full-dose with Alum vs Full-dose without Alum	1·10	0·54-2·23	0·79	0·96	0·48-1·92	0·90
	Low-dose with Alum vs Low-dose without Alum	1·86	0·67-5·19	0·23	1·19	0·46-3·11	0·70

Anti-O:2

Timepoint	Pairwise comparison	Unadjusted ratios			Adjusted ratios		
		Unadjusted GMR	95% CI	p-value	Adjusted GMR	95% CI	p-value
D29	Full-dose with Alum vs Full-dose without Alum	0·16	0·06-0·43	0·0003	0·16	0·07-0·35	<0·0001
	Low-dose with Alum vs Low-dose without Alum	0·13	0·04-0·52	0·0040	0·17	0·06-0·49	0·0020
D169	Full-dose with Alum vs Full-dose without Alum	0·30	0·11-0·81	0·018	0·23	0·11-0·52	0·0007
	Low-dose with Alum vs Low-dose without Alum	0·42	0·09-1·99	0·27	0·34	0·12-1·02	0·055
D176	Full-dose with Alum vs Full-dose without Alum	0·39	0·16-0·95	0·039	0·27	0·12-0·60	0·0020
	Low-dose with Alum vs Low-dose without Alum	0·43	0·12-1·53	0·19	0·35	0·12-1·04	0·058
D197	Full-dose with Alum vs Full-dose without Alum	0·41	0·17-0·97	0·043	0·29	0·13-0·66	0·0036
	Low-dose with Alum vs Low-dose without Alum	0·54	0·15-1·94	0·34	0·45	0·15-1·35	0·15

Anti-*Salmonella* Paratyphi A

Timepoint	Pairwise comparison	Unadjusted ratios			Adjusted ratios		
		Unadjusted GMR	95% CI	p-value	Adjusted GMR	95% CI	p-value
D29	Full-dose with Alum vs Full-dose without Alum	0·35	0·17-0·72	0·0046	0·43	0·22-0·84	0·014
	Low-dose with Alum vs Low-dose without Alum	0·48	0·18-1·31	0·15	0·43	0·16-1·17	0·094
D169	Full-dose with Alum vs Full-dose without Alum	0·53	0·28-1·01	0·052	0·59	0·30-1·17	0·13
	Low-dose with Alum vs Low-dose without Alum	0·65	0·24-1·76	0·39	0·55	0·20-1·54	0·24
D176	Full-dose with Alum vs Full-dose without Alum	0·46	0·24-0·91	0·027	0·59	0·30-1·18	0·13
	Low-dose with Alum vs Low-dose without Alum	0·63	0·24-1·66	0·35	0·54	0·19-1·51	0·23
D197	Full-dose with Alum vs Full-dose without Alum	0·49	0·24-1·02	0·057	0·57	0·29-1·15	0·12
	Low-dose with Alum vs Low-dose without Alum	0·87	0·30-2·52	0·80	0·53	0·19-1·48	0·21

Alum, aluminium hydroxide; CI, confidence interval; D, day; GMR, geometric mean ratio; IgG, immunoglobulin G

Anti-Vi-specific IgG antibodies in serum were measured by a qualified, fit-for-purpose enzyme-linked immunosorbent assay (ELISA); results were expressed in µg/mL. Anti-O:2 IgG antibodies in serum were measured by an ELISA which used a standard curve with an assigned value of ELISA units (EU)/mL using the O:2 as coating antigen; results were expressed in EU/mL. Serum bactericidal activity against *Salmonella* Paratyphi A was measured by serum bactericidal assay. Results were expressed in serum titers, defined as serum dilutions giving 50·0% inhibition of bacterial growth (IC_{50}).

Unadjusted results are based on a linear model for a visit with fixed effect for treatment. Adjusted results are based on a linear mixed model with fixed effect for treatment, timepoint, baseline value, treatment and timepoint interaction, and repeated timepoint effect within a participant under an exchangeable covariance matrix. The model assumes unequal variances between treatment groups.

Supplementary table S11. Anti-O:2 IgG antibody GMCs, GMRs, and seroresponse rates as measured by ELISA (Per-Protocol Set)

	Low-dose without Alum			Low-dose with Alum			Full-dose without Alum			Full-dose with Alum			Control		
	N		Value (95% CI)	N		Value (95% CI)	N		Value (95% CI)	N		Value (95% CI)	N		Value (95% CI)
**GMC (EU/mL)**															
D1	12		36·35 (15·21-86·89)	11		28·31 (11·29-70·97)	21		25·28 (14·73-43·39)	24		25·90 (13·56-49·44)	24		22·15 (13·18-37·23)
D29	12		4150·79 (1362·97-12640·82)	11		559·62 (177·08-1768·50)	21		4110·58 (1975·76-8552·10)	24		668·30 (314·08-1422·01)	24		28·19 (16·07-49·47)
D169	8		702·49 (138·39-3565·88)	10		298·02 (85·01-1044·76)	21		894·08 (397·22-2012·40)	22		268·09 (124·74-576·18)	21		18·43 (10·91-31·13)
D176	8		1079·75 (322·08-3619·77)	10		464·70 (188·39-1146·25)	18		1342·38 (644·96-2793·95)	18		522·60 (264·74-1031·60)	19		18·24 (10·38-32·05)
D197	8		958·21 (298·69-3074·02)	8		520·77 (167·69-1617·23)	16		956·89 (440·21-2080·03)	18		388·50 (219·98-686·13)	20		17·28 (10·13-29·47)
**GMR**															
D29	12		114·19 (44·83-290·86)	11		19·77 (10·03-38·96)	21		162·61 (91·17-290·04)	24		25·81 (13·15-50·64)	24		1·27 (1·02-1·60)
D169	8		26·15 (6·12-111·64)	10		9·11 (3·83-21·64)	21		37·28 (20·47-67·89)	22		9·15 (4·52-18·51)	21		0·92 (0·80-1·07)
D176	8		40·19 (11·79-136·94)	10		14·20 (7·11-28·38)	18		68·01 (39·46-117·23)	18		19·12 (8·97-40·76)	19		0·86 (0·77-0·96)
D197	8		35·66 (10·36-122·82)	8		19·23 (9·58-38·60)	16		51·16 (27·32-95·79)	18		17·86 (9·42-33·86)	20		0·95 (0·81-1·12)
**Percentage of participants with at least 4-fold anti-O:2 IgG increase from baseline**															
	**N**	**n**	**% (95% CI)**	**N**	**n**	**% (95% CI)**	**N**	**n**	**% (95% CI)**	**N**	**n**	**% (95% CI)**	**N**	**n**	**% (95% CI)**
D29	12	12	100 (73·5-100)	11	9	81·8 (48·2-97·7)	21	21	100 (83·9-100)	24	20	83·3 (62·6-95·3)	24	1	4·2 (0·1-21·1)
D169	8	7	87·5 (47·3-99·7)	10	7	70·0 (34·8-93·3)	21	20	95·2 (76·2-99·9)	22	16	72·7 (49·8-89·3)	21	0	0·0 (0·0-16·1)
D176	8	8	100 (63·1-100)	10	8	80·0 (44·4-97·5)	18	18	100 (81·5-100)	18	15	83·3 (58·6-96·4)	19	0	0·0 (0·0-17·6)
D197	8	8	100 (63·1-100)	8	7	87·5 (47·3-99·7)	16	15	93·8 (69·8-99·8)	18	16	88·9 (65·3-98·6)	20	0	0·0 (0·0-16·8)

Alum, aluminium hydroxide; CI, confidence interval; D, day; ELISA, enzyme-linked immunosorbent assay; EU, ELISA units; GMC, geometric mean concentration; GMR, within-participant geometric mean ratio comparing geometric mean to D1 pre-dose; IgG, immunoglobulin G; N, number of participants with available results in each category; n, number of participants with ≥4-fold increase from baseline in anti-O:2 antibodies

The Per-Protocol Set was defined by timepoint.

Supplementary figure S6. Reverse cumulative distribution curves of anti-O:2 IgG by timepoint (Per-Protocol Set)

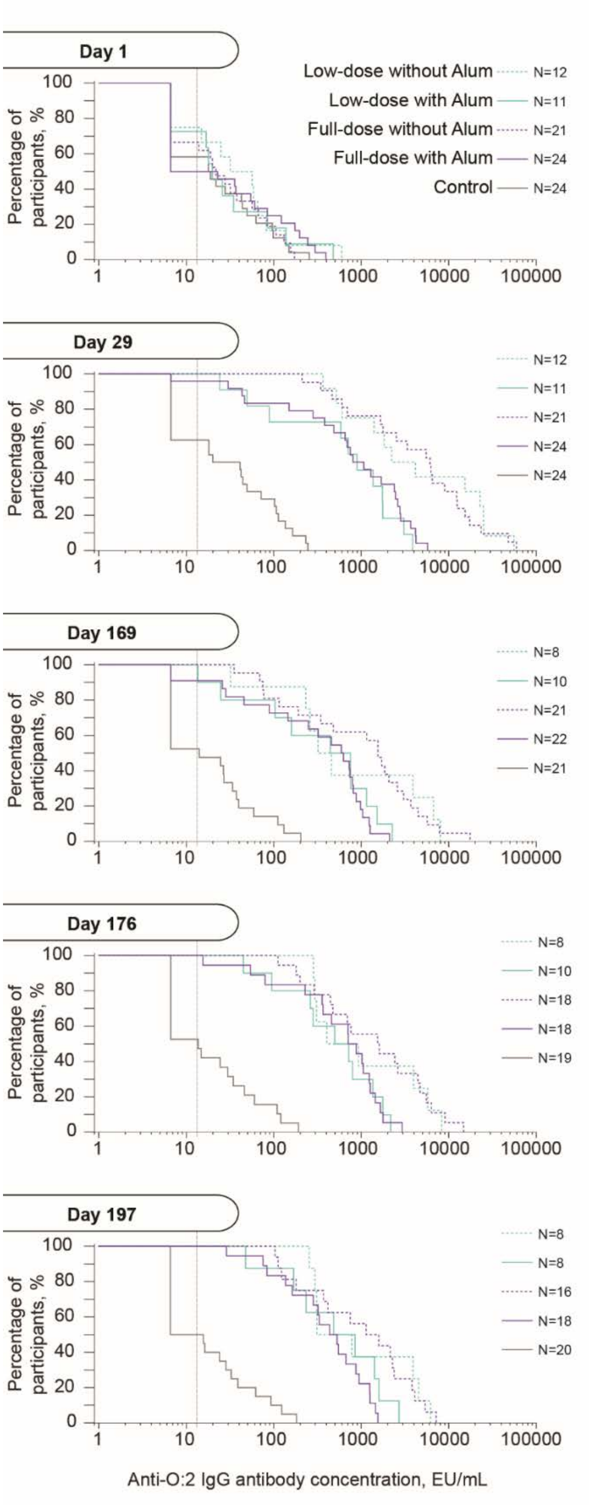

Alum, aluminium hydroxide; EU, ELISA units; IgG, immunoglobulin G; N, number of participants
The dashed line represents the lower limit of quantification for anti-O:2 IgG (13·3 EU/mL).
The Per-Protocol Set was defined by timepoint.

Supplementary table S12. GMTs, GMRs, and percentage of participants with at least 4-fold increase from baseline for bactericidal antibodies against *Salmonella* Paratyphi A as measured by serum bactericidal assay (Per-Protocol Set)

	Low-dose without Alum			Low-dose with Alum			Full-dose without Alum			Full-dose with Alum			Control		
	**N**		**Value (95% CI)**	**N**		**Value (95% CI)**	**N**		**Value (95% CI)**	**N**		**Value (95% CI)**	**N**		**Value (95% CI)**
**GMT (IC_{50})**															
D1	12		161·0 (93·7-276·8)	11		191·4 (106·2-344·8)	21		189·8 (120·1-299·9)	24		143·4 (95·8-214·5)	24		216·7 (143·4-327·4)
D29	12		3615·0 (1694·1-7714·0)	11		1738·7 (708·5-4266·7)	21		3621·2 (2188·0-5993·0)	24		1268·9 (673·2-2391·5)	24		233·4 (162·8-334·6)
D169	8		1068·8 (691·0-1653·4)	10		692·6 (380·3-1261·4)	21		1328·1 (759·6-2322·3)	22		701·9 (395·5-1245·7)	21		166·2 (115·3-239·4)
D176	8		1392·0 (677·7-2859·3)	10		883·4 (478·9-1629·5)	18		2239·0 (1276·5-3927·1)	18		1038·1 (568·7-1895·0)	19		149·4 (100·8-221·3)
D197	8		1303·6 (748·7-2269·6)	8		1134·8 (681·7-1888·9)	16		1609·5 (825·6-3137·7)	18		793·9 (413·6-1523·9)	20		156·3 (103·2-236·7)
**GMR**															
D29	12		22·45 (8·22-61·27)	11		9·09 (3·58-23·06)	21		19·08 (10·61-34·32)	24		8·85 (5·20-15·06)	24		1·08 (0·90-1·30)
D169	8		5·43 (2·29-12·87)	10		3·42 (1·74-6·73)	21		7·87 (4·67-13·24)	22		5·17 (3·28-8·16)	21		0·84 (0·63-1·13)
D176	8		7·08 (2·21-22·62)	10		4·37 (2·08-9·16)	18		13·17 (7·76-22·33)	18		6·86 (4·02-11·71)	19		0·81 (0·60-1·08)
D197	8		6·63 (2·55-17·23)	8		5·15 (2·69-9·85)	16		11·08 (6·14-20·02)	18		6·43 (3·87-10·69)	20		0·82 (0·61-1·12)
**Percentage of participants with at least 4-fold increase from baseline**															
	**N**	**n**	**% (95% CI)**	**N**	**n**	**% (95% CI)**	**N**	**n**	**% (95% CI)**	**N**	**n**	**% (95% CI)**	**N**	**n**	**% (95% CI)**
D29	12	11	91·7 (61·5-99·8)	11	8	72·7 (39·0-94·0)	21	19	90·5 (69·6-98·8)	24	18	75·0 (53·3-90·2)	24	0	0·0 (0·0-14·2)
D169	8	4	50·0 (15·7-84·3)	10	4	40·0 (12·2-73·8)	21	16	76·2 (52·8-91·8)	22	13	59·1 (36·4-79·3)	21	0	0·0 (0·0-16·1)
D176	8	4	50·0 (15·7-84·3)	10	6	60·0 (26·2-87·8)	18	16	88·9 (65·3-98·6)	18	11	61·1 (35·7-82·7)	19	0	0·0 (0·0-17·6)
D197	8	4	50·0 (15·7-84·3)	8	6	75·0 (34·9-96·8)	16	14	87·5 (61·7-98·4)	18	13	72·2 (46·5-90·3)	20	0	0·0 (0·0-16·8)

Alum, aluminium hydroxide; CI, confidence interval; D, day; GMR, within-participant geometric mean ratio comparing geometric mean to D1 pre-dose; GMT, geometric mean titer; IC_{50}, 50% inhibition of bacterial growth; N, number of participants with available results in each category; n, number of participants with ≥4-fold increase from baseline in bactericidal antibodies against *Salmonella* Paratyphi A

The Per-Protocol Set was defined by timepoint.

Supplementary figure S7. Reverse cumulative distribution curves of bactericidal antibodies against *Salmonella* Paratyphi A (as measured by SBA) by treatment group (Per-Protocol Set)

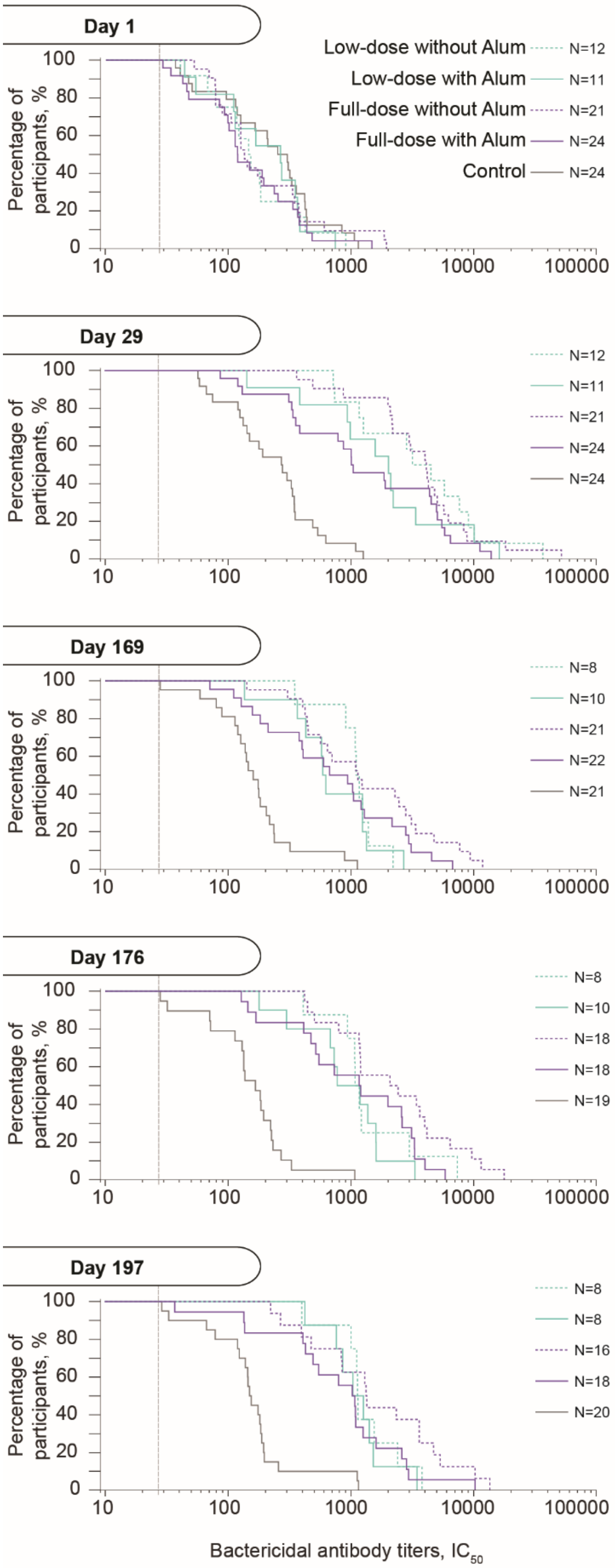

Alum, aluminium hydroxide; IC_{50}, 50% inhibition of bacterial growth; SBA, serum bactericidal assay; N, number of participants

The dashed line represents lower limit of quantification for bactericidal antibodies against *Salmonella* Paratyphi A (27·3 IC_{50}). The Per-Protocol Set was defined by timepoint.

Supplementary table S13. Anti-Vi IgG antibody GMCs, GMRs, and seroresponse rates as measured by ELISA (Full Analysis Set)

	Low-dose without Alum			Low-dose with Alum			Full-dose without Alum			Full-dose with Alum			Control		
	**N**		**Value (95% CI)**	**N**		**Value (95% CI)**	**N**		**Value (95% CI)**	**N**		**Value (95% CI)**	**N**		**Value (95% CI)**
**GMC (µg/mL)**															
D1	12		1·10 (-)	11		1·52 (0·93-2·48)	22		1·14 (1·06-1·23)	24		1·17 (1·03-1·33)	24		1·10 (-)
D29	12		14·12 (8·21-24·29)	11		19·61 (9·10-42·28)	22		65·54 (38·23-112·35)	24		36·97 (20·56-66·48)	24		4·95 (3·23-7·59)
D169	8		4·36 (2·01-9·47)	11		11·30 (5·31-24·02)	21		18·58 (9·97-34·60)	22		12·55 (7·06-22·32)	21		6·18 (3·50-10·92)
D176	8		10·90 (4·72-25·21)	10		18·78 (9·05-38·97)	20		40·29 (22·93-70·80)	21		52·38 (36·54-75·10)	21		5·00 (3·00-8·32)
D197	8		10·16 (5·08-20·30)	10		20·23 (10·72-38·18)	21		31·78 (19·21-52·57)	22		37·63 (26·14-54·16)	21		4·80 (2·92-7·90)
**GMR**															
D29	12		12·84 (7·46-22·08)	11		12·90 (6·77-24·58)	22		57·50 (34·51-95·80)	24		31·55 (18·74-53·11)	24		4·50 (2·93-6·90)
D169	8		3·96 (1·82-8·61)	11		7·43 (3·93-14·04)	21		16·27 (8·94-29·61)	22		10·65 (6·59-17·22)	21		5·62 (3·18-9·93)
D176	8		9·91 (4·29-22·92)	10		11·96 (6·05-23·65)	20		35·23 (20·34-61·01)	21		44·31 (34·25-57·33)	21		4·54 (2·73-7·57)
D197	8		9·23 (4·62-18·46)	10		12·89 (6·93-23·96)	21		27·84 (17·09-45·35)	22		31·93 (24·94-40·88)	21		4·37 (2·66-7·18)
**Seroresponse*: participants with anti-Vi IgG concentration ≥4·3 µg/mL**															
	**N**	**n**	**% (95% CI)**	**N**	**n**	**% (95% CI)**	**N**	**n**	**% (95% CI)**	**N**	**n**	**% (95% CI)**	**N**	**n**	**% (95% CI)**
D1	12	0	0·0 (0·0-26·5)	9	0	0·0 (0·0-33·6)	22	0	0·0 (0·0-15·4)	23	0	0·0 (0·0-14·8)	24	0	0·0 (0·0-14·2)
D29	12	12	100 (73·5-100)	9	8	88·9 (51·8-99·7)	22	22	100 (84·6-100)	23	22	95·7 (78·1-99·9)	24	13	54·2 (32·8-74·4)
D169	8	6	75·0 (34·9-96·8)	9	8	88·9 (51·8-99·7)	21	20	95·2 (76·2-99·9)	21	17	81·0 (58·1-94·6)	21	13	61·9 (38·4-81·9)
D176	8	6	75·0 (34·9-96·8)	8	7	87·5 (47·3-99·7)	20	19	95·0 (75·1-99·9)	20	20	100 (83·2-100)	21	12	57·1 (34·0-78·2)
D197	8	7	87·5 (47·3-99·7)	8	7	87·5 (47·3-99·7)	21	20	95·2 (76·2-99·9)	21	21	100 (83·9-100)	21	12	57·1 (34·0-78·2)
**Seroresponse*: participants with anti-Vi IgG concentration ≥2·0 µg/mL**															
D1	12	0	0·0 (0·0-26·5)	9	0	0·0 (0·0-33·6)	21	0	0·0 (0·0-16·1)	23	0	0·0 (0·0-14·8)	24	0	0·0 (0·0-14·2)
D29	12	12	100 (73·5-100)	9	9	100 (66·4-100)	21	21	100 (83·9-100)	23	22	95·7 (78·1-99·9)	24	20	83·3 (62·6-95·3)
D169	8	6	75·0 (34·9-96·8)	9	8	88·9 (51·8-99·7)	20	19	95·0 (75·1-99·9)	21	21	100 (83·9-100)	21	17	81·0 (58·1-94·6)
D176	8	8	100 (63·1-100)	8	8	100 (63·1-100)	19	18	94·7 (74·0-99·9)	20	20	100 (83·2-100)	21	16	76·2 (52·8-91·8)
D197	8	8	100 (63·1-100)	8	8	100 (63·1-100)	20	20	100 (83·2-100)	21	21	100 (83·9-100)	21	16	76·2 (52·8-91·8)

Alum, aluminium hydroxide; CI, confidence interval; D, day; ELISA enzyme-linked immunosorbent assay; GMC, geometric mean concentration; GMR, within-participant geometric mean ratio comparing geometric mean to D1 pre-dose; IgG, immunoglobulin G; N, number of participants with available results in each category; n, number of participants with anti-Vi IgG concentration ≥2·0 µg/mL/≥4·3 µg/mL

*In participants seronegative (anti-Vi IgG antibody concentrations below 4·3 µg/mL or 2·0 µg/mL, respectively for each threshold) at baseline.

The Full Analysis Set was defined by timepoint.

Supplementary table S14. Anti-O:2 IgG antibody GMCs, GMRs, and seroresponse rates as measured by ELISA (Full Analysis Set)

	Low-dose without Alum			Low-dose with Alum			Full-dose without Alum			Full-dose with Alum			Control		
	**N**		**Value (95% CI)**	**N**		**Value (95% CI)**	**N**		**Value (95% CI)**	**N**		**Value (95% CI)**	**N**		**Value (95% CI)**
**GMC (EU/mL)**															
D1	12		36·35 (15·21-86·89)	11		28·31 (11·29-70·97)	22		26·12 (15·56-43·85)	24		25·90 (13·56-49·44)	24		22·15 (13·18-37·23)
D29	12		4150·79 (1362·97-12640·82)	11		559·62 (177·08-1768·50)	22		4363·99 (2151·08-8853·41)	24		668·30 (314·08-1422·01)	24		28·19 (16·07-49·47)
D169	8		702·49 (138·39-3565·88)	11		210·92 (54·28-819·55)	21		894·08 (397·22-2012·40)	22		268·09 (124·74-576·18)	21		18·43 (10·91-31·13)
D176	8		1079·75 (322·08-3619·77)	10		464·70 (188·39-1146·25)	20		1431·82 (740·51-2768·50)	21		545·30 (301·92-984·86)	21		18·42 (10·84-31·30)
D197	8		958·21 (298·69-3074·02)	10		506·97 (213·98-1201·11)	21		1287·77 (660·93-2509·12)	22		494·23 (293·40-832·50)	21		18·60 (10·96-31·58)
**GMR**															
D29	12		114·19 (44·83-290·86)	11		19·77 (10·03-38·96)	22		167·06 (96·11-290·41)	24		25·81 (13·15-50·64)	24		1·27 (1·02-1·60)
D169	8		26·15 (6·12-111·64)	11		7·45 (3·06-18·17)	21		37·28 (20·47-67·89)	22		9·15 (4·52-18·51)	21		0·92 (0·80-1·07)
D176	8		40·19 (11·79-136·94)	10		14·20 (7·11-28·38)	20		65·02 (39·81-106·17)	21		18·40 (9·55-35·45)	21		0·92 (0·78-1·09)
D197	8		35·66 (10·36-122·82)	10		15·50 (8·24-29·13)	21		53·69 (32·80-87·90)	22		16·87 (9·45-30·10)	21		0·93 (0·79-1·09)
**Percentage of participants with at least 4-fold anti-O:2 IgG increase from baseline**															
	**N**	**n**	**% (95% CI)**	**N**	**n**	**% (95% CI)**	**N**	**n**	**% (95% CI)**	**N**	**n**	**% (95% CI)**	**N**	**n**	**% (95% CI)**
D29	12	12	100 (73·5-100)	11	9	81·8 (48·2-97·7)	22	22	100 (84·6-100)	24	20	83·3 (62·6-95·3)	24	1	4·2 (0·1-21·1)
D169	8	7	87·5 (47·3-99·7)	11	7	63·6 (30·8-89·1)	21	20	95·2 (76·2-99·9)	22	16	72·7 (49·8-89·3)	21	0	0·0 (0·0-16·1)
D176	8	8	100 (63·1-100)	10	8	80·0 (44·4-97·5)	20	20	100 (83·2-100)	21	18	85·7 (63·7-97·0)	21	0	0·0 (0·0-16·1)
D197	8	8	100 (63·1-100)	10	9	90·0 (55·5-99·7)	21	20	95·2 (76·2-99·9)	22	19	86·4 (65·1-97·1)	21	0	0·0 (0·0-16·1)

Alum, aluminium hydroxide; CI, confidence interval; D, day; ELISA, enzyme-linked immunosorbent assay; EU, ELISA units; GMC, geometric mean concentration; GMR, within-participant geometric mean ratio comparing geometric mean to D1 pre-dose; IgG, immunoglobulin G; N, number of participants with available results in each category; n, number of participants with ≥4-fold increase from baseline in anti-O:2 antibodies

The Full Analysis Set was defined by timepoint.

Supplementary table S15. GMTs, GMRs, and percentage of participants with at least 4-fold increase from baseline for bactericidal antibodies against *Salmonella* Paratyphi A as measured by serum bactericidal assay (Full Analysis Set)

	Low-dose without Alum			Low-dose with Alum			Full-dose without Alum			Full-dose with Alum			Control		
	**N**		**Value (95% CI)**	**N**		**Value (95% CI)**	**N**		**Value (95% CI)**	**N**		**Value (95% CI)**	**N**		**Value (95% CI)**
**GMT (IC_{50})**															
D1	12		161·0 (93·7-276·8)	11		191·4 (106·2-344·8)	22		188·3 (121·8-291·0)	24		143·4 (95·8-214·5)	24		216·7 (143·4-327·4)
D29	12		3615·0 (1694·1-7714·0)	11		1738·7 (708·5-4266·7)	22		3561·0 (2203·1-5755·7)	24		1268·9 (673·2-2391·5)	24		233·4 (162·8-334·6)
D169	8		1068·8 (691·0-1653·4)	11		669·8 (390·6-1148·6)	21		1328·1 (759·6-2322·3)	22		701·9 (395·5-1245·7)	21		166·2 (115·3-239·4)
D176	8		1392·0 (677·7-2859·3)	10		883·4 (478·9-1629·5)	20		1905·0 (1087·6-3336·7)	21		836·0 (453·1-1542·5)	21		161·7 (108·3-241·6)
D197	8		1303·6 (748·7-2269·6)	10		822·0 (439·7-1536·8)	21		1520·6 (877·7-2634·4)	22		780·7 (435·9-1398·3)	21		162·2 (108·6-242·2)
**GMR**															
D29	12		22·45 (8·22-61·27)	11		9·09 (3·58-23·06)	22		18·91 (10·82-33·05)	24		8·85 (5·20-15·06)	24		1·08 (0·90-1·30)
D169	8		5·43 (2·29-12·87)	11		3·50 (1·91-6·40)	21		7·87 (4·67-13·24)	22		5·17 (3·28-8·16)	21		0·84 (0·63-1·13)
D176	8		7·08 (2·21-22·62)	10		4·37 (2·08-9·16)	20		11·75 (7·13-19·36)	21		6·07 (3·70-9·95)	21		0·82 (0·63-1·07)
D197	8		6·63 (2·55-17·23)	10		4·06 (2·12-7·81)	21		9·01 (5·53-14·68)	22		5·75 (3·60-9·18)	21		0·82 (0·62-1·10)
**Percentage of participants with at least 4-fold increase from baseline**															
	**N**	**n**	**% (95% CI)**	**N**	**n**	**% (95% CI)**	**N**	**n**	**% (95% CI)**	**N**	**n**	**% (95% CI)**	**N**	**n**	**% (95% CI)**
D29	12	11	91·7 (61·5-99·8)	11	8	72·7 (39·0-94·0)	22	20	90·9 (70·8-98·9)	24	18	75·0 (53·3-90·2)	24	0	0·0 (0·0-14·2)
D169	8	4	50·0 (15·7-84·3)	11	5	45·5 (16·7-76·6)	21	16	76·2 (52·8-91·8)	22	13	59·1 (36·4-79·3)	21	0	0·0 (0·0-16·1)
D176	8	4	50·0 (15·7-84·3)	10	6	60·0 (26·2-87·8)	20	17	85·0 (62·1-96·8)	21	12	57·1 (34·0-78·2)	21	0	0·0 (0·0-16·1)
D197	8	4	50·0 (15·7-84·3)	10	6	60·0 (26·2-87·8)	21	18	85·7 (63·7-97·0)	22	14	63·6 (40·7-82·8)	21	0	0·0 (0·0-16·1)

Alum, aluminium hydroxide; CI, confidence interval; D, day; GMR, within-participant geometric mean ratio comparing geometric mean to D1 pre-dose; GMT, geometric mean titer; IC_{50}, 50% inhibition of bacterial growth; N, number of participants with available results in each category; n, number of participants with ≥4-fold increase from baseline in bactericidal antibodies against *Salmonella* Paratyphi A

The Full Analysis Set was defined by timepoint.

Supplementary figure S8. Graphical abstract

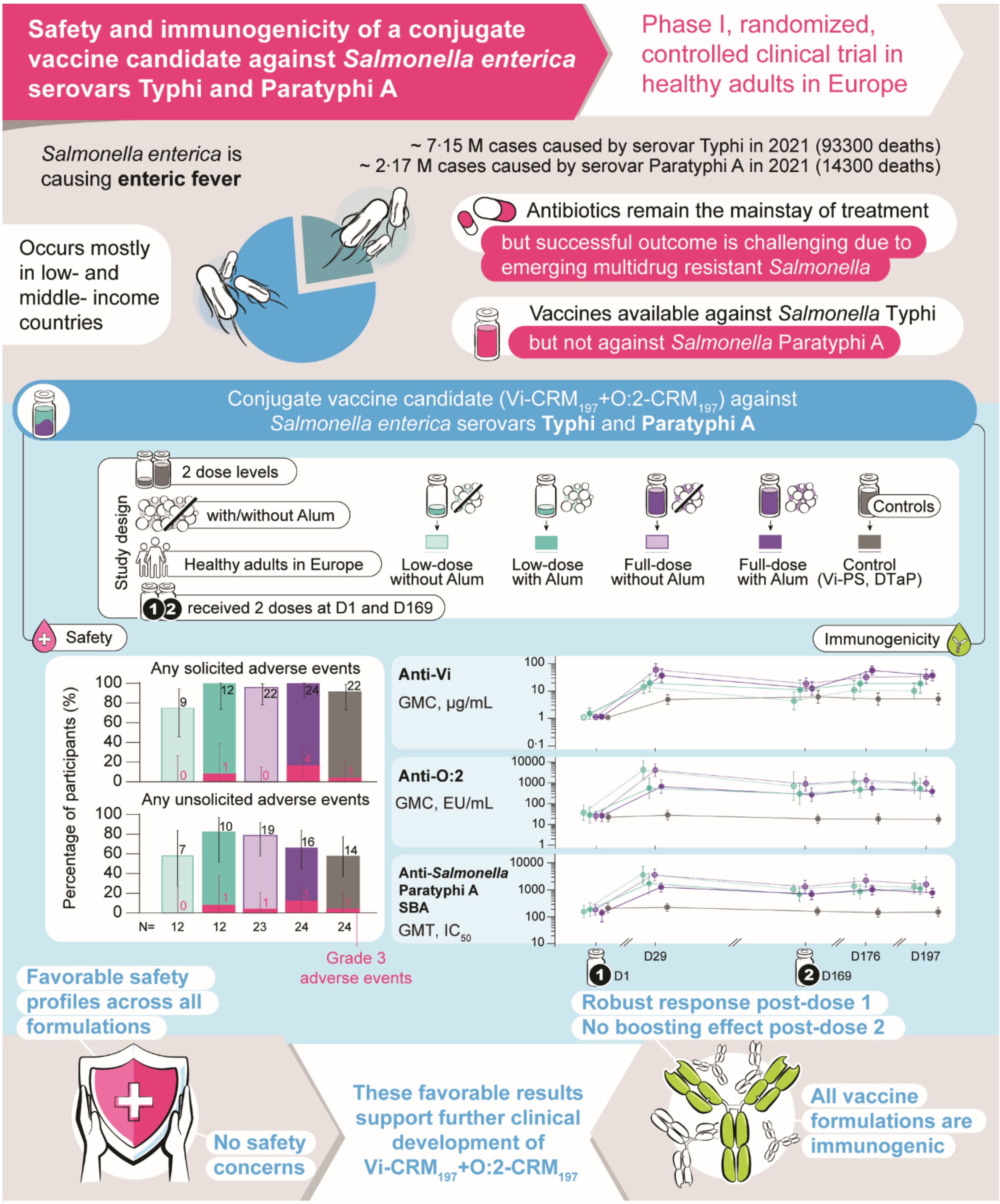

Alum, aluminium hydroxide; CRM197, Cross-Reacting Material 197; D, day; DTaP, diphtheria toxoid-tetanus toxoid-acellular pertussis vaccine; GMC, geometric mean concentration; GMT, geometric mean titer; IC50, 50% inhibition of bacterial growth; M, million; SBA, serum bactericidal assay; Vi-PS, typhoid Vi polysaccharide vaccine.

The study protocol (including the protocol amendments) and the statistical analysis plan are available at https://www.gsk-studyregister.com/en/trial-details/?id=205480#documents-section.

Supplementary references

1. Food and Drug Administration. Guidance for Industry. Toxicity Grading Scale for Healthy Adult and Adolescent Volunteers Enrolled in Preventive Vaccine Clinical Trials. 2007. https://www.fda.gov/media/73679/download (accessed Jan 28, 2025).
2. Carducci M, Massai L, Lari E, et al. Qualification of an enzyme-linked immunosorbent assay for quantification of anti-Vi IgG in human sera. *Front Immunol* 2024; **15**: 1466869.
3. Carducci M, Massai L, Lari E, et al. Development and characterization of high-throughput serological assays to measure magnitude and functional immune response against *S*. Paratyphi A in human samples. *Front Immunol* 2024; **15**: 1443137.
4. Szu SC, Hunt S, Xie G, et al. A human IgG anti-Vi reference for *Salmonella* typhi with weight-based antibody units assigned. *Vaccine* 2013; **31**: 1970–4.
5. Thuluva S, Paradkar V, Matur R, Turaga K, Gv SR. A multicenter, single-blind, randomized, phase-2/3 study to evaluate immunogenicity and safety of a single intramuscular dose of biological E's Vi-capsular polysaccharide-CRM_{197} conjugate typhoid vaccine (TyphiBEV™) in healthy infants, children, and adults in comparison with a licensed comparator. *Hum Vaccin Immunother* 2022; **18**: 2043103.
6. Szu SC, Klugman KP, Hunt S. Re-examination of immune response and estimation of anti-Vi IgG protective threshold against typhoid fever-based on the efficacy trial of Vi conjugate in young children. *Vaccine* 2014; **32**: 2359–63.

Section/topic	No	CONSORT 2025 checklist item description	Reported on page no.
**Title and abstract**			
Title and structured abstract	1a	Identification as a randomised trial	1
	1b	Structured summary of the trial design, methods, results, and conclusions	1
**Open science**			
Trial registration	2	Name of trial registry, identifying number (with URL) and date of registration	3
Protocol and statistical analysis plan	3	Where the trial protocol and statistical analysis plan can be accessed	3
Data sharing	4	Where and how the individual de-identified participant data (including data dictionary), statistical code and any other materials can be accessed	3
Funding and conflicts of interest	5a	Sources of funding and other support (eg, supply of drugs), and role of funders in the design, conduct, analysis and reporting of the trial	6, 12
	5b	Financial and other conflicts of interest of the manuscript authors	12
**Introduction**			
Background and rationale	6	Scientific background and rationale	2
Objectives	7	Specific objectives related to benefits and harms	2
**Methods**			
Patient and public involvement	8	Details of patient or public involvement in the design, conduct and reporting of the trial	-
Trial design	9	Description of trial design including type of trial (eg, parallel group, crossover), allocation ratio, and framework (eg, superiority, equivalence, non-inferiority, exploratory)	3
Changes to trial protocol	10	Important changes to the trial after it commenced including any outcomes or analyses that were not prespecified, with reason	NA
Trial setting	11	Settings (eg, community, hospital) and locations (eg, countries, sites) where the trial was conducted	3
Eligibility criteria	12a	Eligibility criteria for participants	3 (+appendix)
	12b	If applicable, eligibility criteria for sites and for individuals delivering the interventions (eg, surgeons, physiotherapists)	NA
Intervention and comparator	13	Intervention and comparator with sufficient details to allow replication. If relevant, where additional materials describing the intervention and comparator (eg, intervention manual) can be accessed	4
Outcomes	14	Prespecified primary and secondary outcomes, including the specific measurement variable (eg, systolic blood pressure), analysis metric (eg, change from baseline, final value, time to event), method of aggregation (eg, median, proportion), and time point for each outcome	4
Harms	15	How harms were defined and assessed (eg, systematically, non-systematically)	4,6 (+appendix)
Sample size	16a	How sample size was determined, including all assumptions supporting the sample size calculation	6 (+appendix)
	16b	Explanation of any interim analyses and stopping guidelines	3 (+appendix)
Randomisation:			
Sequence generation	17a	Who generated the random allocation sequence and the method used	3
	17b	Type of randomisation and details of any restriction (eg, stratification, blocking and block size)	3
Allocation concealment mechanism	18	Mechanism used to implement the random allocation sequence (eg, central computer/telephone; sequentially numbered, opaque, sealed containers), describing any steps to conceal the sequence until interventions were assigned	3
Implementation	19	Whether the personnel who enrolled and those who assigned participants to the interventions had access to the random allocation sequence	3 (+appendix)
Blinding	20a	Who was blinded after assignment to interventions (eg, participants, care providers, outcome assessors, data analysts)	3-4

	20b	If blinded, how blinding was achieved and description of the similarity of interventions	3
Statistical methods	21a	Statistical methods used to compare groups for primary and secondary outcomes, including harms	6 (+appendix)
	21b	Definition of who is included in each analysis (eg, all randomised participants), and in which group	6 (+appendix)
	21c	How missing data were handled in the analysis	-
	21d	Methods for any additional analyses (eg, subgroup and sensitivity analyses), distinguishing prespecified from post hoc	6
**Results**			
Participant flow, including flow diagram	22a	For each group, the numbers of participants who were randomly assigned, received intended intervention, and were analysed for the primary outcome	5-6 (+appendix)
	22b	For each group, losses and exclusions after randomisation, together with reasons	5
Recruitment	23a	Dates defining the periods of recruitment and follow-up for outcomes of benefits and harms	6
	23b	If relevant, why the trial ended or was stopped	NA
Intervention and comparator delivery	24a	Intervention and comparator as they were actually administered (eg, where appropriate, who delivered the intervention/comparator, how participants adhered, whether they were delivered as intended (fidelity))	-
	24b	Concomitant care received during the trial for each group	NA
Baseline data	25	A table showing baseline demographic and clinical characteristics for each group	6
Numbers analysed, outcomes and estimation	26	For each primary and secondary outcome, by group:<br>● the number of participants included in the analysis<br>● the number of participants with available data at the outcome time point<br>● result for each group, and the estimated effect size and its precision (such as 95% confidence interval)<br>● for binary outcomes, presentation of both absolute and relative effect size	7-10 (+appendix)
Harms	27	All harms or unintended events in each group	7-8 (+appendix)
Ancillary analyses	28	Any other analyses performed, including subgroup and sensitivity analyses, distinguishing pre-specified from post hoc	9-10 (+appendix)
**Discussion**			
Interpretation	29	Interpretation consistent with results, balancing benefits and harms, and considering other relevant evidence	10-12
Limitations	30	Trial limitations, addressing sources of potential bias, imprecision, generalisability, and, if relevant, multiplicity of analyses	11
